# Exploring aortic stiffness in aging mice: a comprehensive methodological overview

**DOI:** 10.18632/aging.206168

**Published:** 2024-12-02

**Authors:** Laetitia Vanalderwiert, Auberi Henry, Juliana Martins de Souza E Silva, Daniel Carvajal-Berrio, Laurent Debelle, Amandine Wahart, Julia Marzi, Katja Schenke-Layland, Gilles Faury, Isabelle Six, Christian E.H. Schmelzer, Jürgen Brinckmann, Heiko Steenbock, Sébastien Almagro, Frédéric Delacoux, Stéphane Jaisson, Philippe Gillery, Pascal Maurice, Hervé Sartelet, Amar Bennasroune, Laurent Duca, Béatrice Romier, Sébastien Blaise

**Affiliations:** 1 UMR CNRS 7369 MEDyC, University of Reims Champagne-Ardenne, Reims 51100, France; 2 Fraunhofer Institute for Microstructure of Materials and Systems IMWS, Halle (Saale) 06120, Germany; 3 Department for Medical Technologies and Regenerative Medicine, Institute of Biomedical Engineering, Eberhard Karls University Tübingen, Tübingen 72076, Germany; 4 Institute of Pharmacy, Faculty of Natural Sciences I, Martin Luther University Halle-Wittenberg, Halle (Saale) 06120, Germany; 5 NMI Natural and Medical Sciences Institute, Reutlingen 72770, Germany; 6 Department of Medicine/Cardiology, Cardiovascular Research Laboratories, David Geffen School of Medicine at UCLA, Los Angeles, CA 90095, USA; 7 INSERM, CHU Grenoble Alpes, University of Grenoble Alpes, Grenoble 38000, France; 8 Research Unit 7517, Pathophysiological Mechanisms and Consequences of Cardiovascular Calcifications (MP3CV), University of Picardie Jules Verne, Amiens, France; 9 Department of Biochemistry, Hospital of Reims, Reims, France; 10 Institute of Virology and Cell Biology, University of Lübeck, Lübeck, Germany; 11 Department of Dermatology, University of Lübeck, Lübeck, Germany; 12 International coordinator of The Exact and Natural Faculty of Reims, University of Reims Champagne-Ardenne, Reims 51100, France

**Keywords:** aortic stiffness, extracellular matrix remodeling, elastic fibers, collagen fibers, ageing, methodological process

## Abstract

Stiffening of the vascular network is associated with the early stages of vascular aging, leading to cardiovascular disorders (hypertension), renal failures, or neurodegenerative diseases (Alzheimer’s). Unfortunately, many people remain undiagnosed because diagnostic methods are either unsuitable for a large population or unfamiliar to clinicians which favor the hypertension evaluation. In preclinical research, stiffness studies are often partially conducted. We think that the evaluation of aortic stiffness is essential as it would improve our understanding of aging diseases progression. We propose here a systematic method using decision trees in a multi-scale and multimodal approaches. Our method was evaluated by analyzing the aortic situation in old and young mice. We demonstrate that both the endothelial and smooth muscle cells exhibit pronounced functional alterations in favor of constriction. Additionally, there is significant remodeling of the extracellular matrix, leading to a drastic degradation of elastic fibers and the accumulation of collagen in the aortic wall. This series of changes contributes to the development of vascular rigidity, a preliminary stage of arterial hypertension. Our results suggest that our method should improve preclinical understanding and encourage clinicians to equip themselves with tools for assessing vascular function, as it is an essential issue for preventing numerous pathologies.

## INTRODUCTION

According to a recent study [1], adults aged 30 to 79 with arterial hypertension (AH) have increased from 650 million to 1.28 billion in 30 years. Nearly half of these individuals are unaware of their condition, primarily due to population growth, metabolic diseases (e.g., obesity, diabetes), and aging. AH significantly raises the risk of heart, brain, or kidney diseases, making it a leading global cause of mortality and morbidity [2]. Thus, adopting a total risk approach for early detection and effective management of AH could prevent cardiovascular complications.

Several studies [3, 4] suggest that the rise in blood pressure is only one symptom of an underlying cardiovascular disease and does not predict its course, except for severe AH. Systolic and diastolic pressures are important indicators but alone cannot reveal cellular or extracellular alterations in organs [5]. The duration of moderately high blood pressure values may play a role in organ functioning changes, making it essential to identify predictive parameters for cardiovascular risk. Some studies propose arterial stiffening measurement as a better parameter to predict risk and mortality in obese, diabetic, or aged patients [6–10].

Vascular stiffening results from vascular wall remodeling, including cellular and extracellular matrix (ECM) alterations [11]. The cellular compartment (vascular smooth muscle cells, SMC, and/or endothelial cells, EC) affects vasocontraction and vasodilation, while extracellular components like elastic and collagen fibers contribute to arterial mechanical properties. Collagen ensures vessel integrity and resistance to stretching [12], and elastic fibers provide flexibility and extensibility while allowing vessel retraction. With age, cellular composition and functionality change [13], and the ECM scaffold undergoes strong remodeling (elastic fiber degradation, collagen neo-synthesis).

To prevent cardiovascular issues, determining a patient’s arterial age is crucial. In this context, the index of arterial stiffening may accurately estimate cardiovascular aging. Various methods are used, in hospitals or by medical practitioners, to measure arterial stiffness (i.e., magnetic resonance imaging (MRI), ultrasound). But others, requiring an invasive approach (i.e., atomic force microscopy (AFM), vascular reactivity), are for experimental research only. The method we present here was designed and tested to evaluate vascular aging in a mouse model. Its accuracy relies on decision trees from both functional and anatomical perspectives.

## RESULTS

### A functional approach to study aortic stiffness

**Figure 1:**
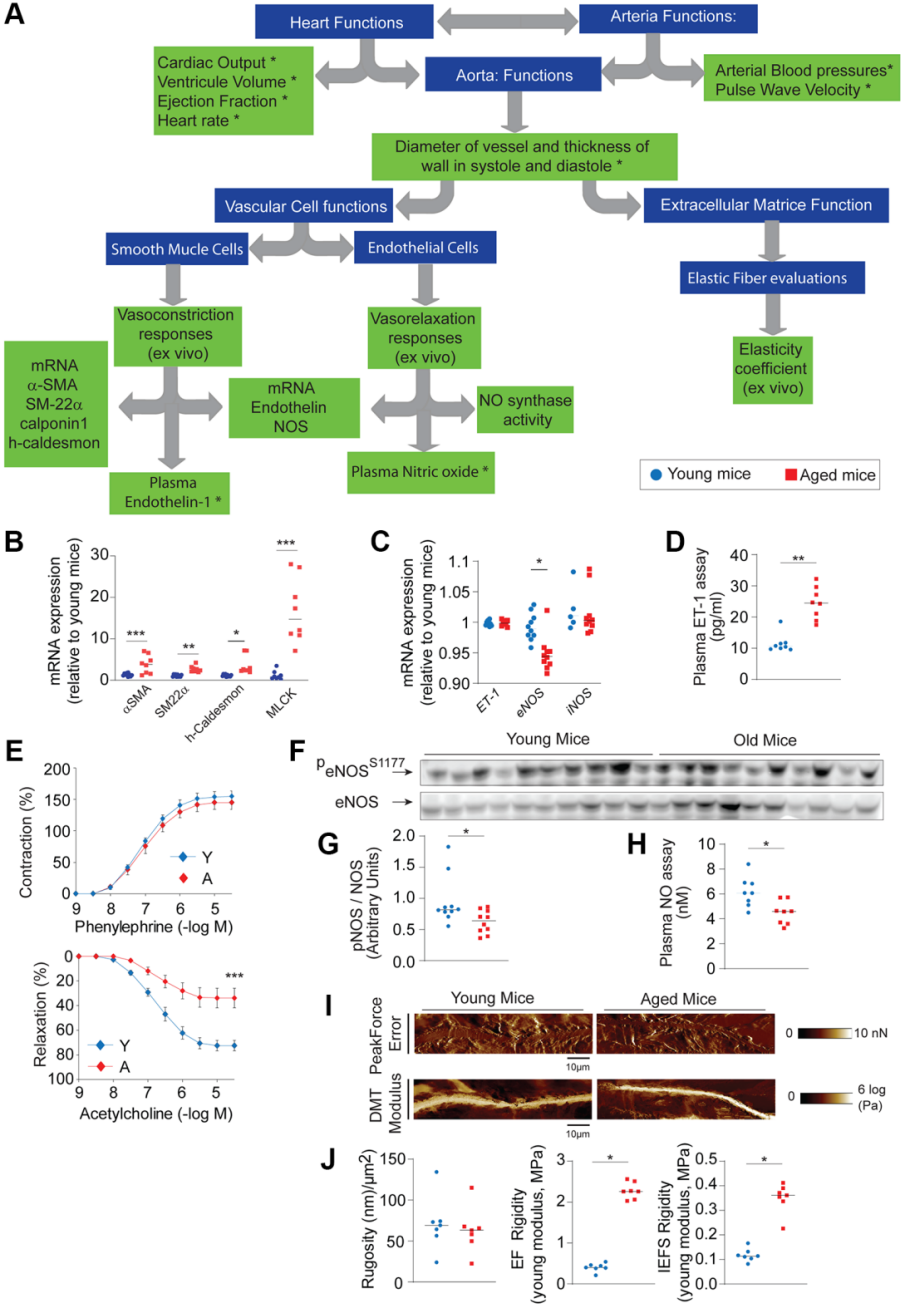
**Functional evaluation of aortic stiffness between old (*n* = 5–10, red) and young (*n* = 5–10, blue) mice.** (**A**) Decision tree allowing the functional evaluation of the animal with the elastic fiber. “^*^” identifies parameters that are methodologically accessible for clinical studies. (**B**) mRNA expression of vascular contraction markers (αSMA, Alpha Smooth Muscle Actin - SM22α, Smooth muscle protein 22-alpha - MLCK, Myosin light-chain kinase). (**C**) mRNA expression of endothelin (ET-1) and endothelial (eNOS) and inducible (iNOS) nitric oxide synthase. (**D**) Plasma assay of endothelin 1. (**E**) *Ex vivo* measurement of vascular reactivity after stimulation with phenylephrine or acetylcholine. (**F**, **G**) Western blot of native and phosphorylated forms of eNOS (panel **F**) and quantification of gray level by ImageJ (panel **G**). (**H**) Plasma dosage of nitric oxide (NO). (**I**, **J**) Evaluation of extracellular matrix: by atomic force microscopy. (**I**) Imaging of elastic fibers (EF) with PeakForce error and DMT modulus (scale bar: 10 µm). (**J**) Quantifications of EF rugosity (left), stiffening of EF (middle) and inter-EF spaces (IEFS, right). Statistical test: Mann-Whitney. Mean+/− SEM. Significant differences (^*^p < 0.05, ^**^p < 0.001, ^***^p < 0.0001, Mann-Whitney).

The assessment of aortic function and its level of stiffness can be easily conducted using general functional methods (e.g., ultrasound or measurement of arterial pressures) or more specific approaches at the cellular scale (i.e., EC and SMC) or the extracellular scale (elastic fibers) (Figure 1A). To illustrate this, we examined the cardiovascular function of young and old mice. Using high-frequency ultrasound, we obtained various anatomical and functional parameters of the heart and aorta. Cardiac parameters, including end-systolic and end-diastolic volumes, left ventricular mass, cardiac output, fractional area change, and fractional shortening, were significantly altered in the aged mice compared to the young mice, suggesting hypertrophy of the older heart (Table 1). Aged mice exhibited increased systolic and pulse pressures, as well as aortic pulse wave velocity (see Table 1). Ultrasound measurements of aortic anatomical parameters and estimations of local Young’s modulus, compliance, and wall distensibility indicated arterial stiffness in aged mice (see Table 1). Muscle hypercontraction or ECM remodeling could explain the vascular stiffness observed. In comparison to young mice, aged mice displayed elevated production/expression of vasoconstrictor factors, such as tissue expression of α-SMA (smooth muscle actin), SM22a (smooth muscle), h-Caldesmon, and MLCK (Myosin light-chain kinase) (Figure 1B), as well as the expression and secretion of endothelin 1 (ET-1) in the plasma (Figure 1C, 1D).

**Table 1:** Cardiovascular parameters obtained from young and aged mice.

		**Young**	**Aged**	**Mann Whitney**
		**(*n* = 10)**	**(*n* = 10)**	**(*p*-value)**
**VASCULAR PARAMETERS**	Systolic blood pressure (mmHg)	124.2 ± 0.8	139.5 ± 1.9	**0.0002**
	Diastolic blood pressure (mmHg)	48.3 ± 1.1	50.6 ± 1.5	0.1983
	Mean Arterial Pressure (mmHg)	73.6 ± 1.0	80.2 ± 1.6	**0.0011**
	Pulse Pressure (mmHg)	75.9 ± 0.3	88.9 ± 1.0	**0.0002**
	Pulse Wave Velocity (m/s)	0.2 ± 0.01	0.3 ± 0.02	**0.0015**
	Systole Diameter, *Ds* (mm)	1,68 ± 0.03	1.08 ± 0.05	**<0.0001**
	Diastole Diameter, *Dd* (mm)	1.48 ± 0.02	0.92 ± 0.04	**<0.0001**
	Systole Surface, As (mm^2^)	2,21 ± 0.07	0.93 ± 0.08	**<0.0001**
	Diastole Surface (Ad, mm^2^)	1,73 ± 0.06	0.69 ± 0.06	**<0.0001**
	Intima-media thickness (h, mm)	0.13 ± 0.003	0.14 ± 0.004	0.0972
	Local PWV (m/s)	0.87 ± 0.12	4.46 ± 0.75	**0.0013**
	Distensibility (*DC*, MPa^−1^)	203.6 ± 51.1	15.8 ± 7.5	**0.0108**
	Young modulus (E, MPa)	0.09 ± 0.02	1.72 ± 0.50	**0.0172**
	Local pulse pressure (DP, MPa)	0.002 ± 0.001	0.09 ± 0.03	**0.0127**
	Compliance (CC, mm^2^/MPa)	142.3 ± 35.5	6.9 ± 3.3	**0.0085**
**HEART PARAMETERS**	Heart Rate in vigilante mice (bpm)	624.0 ± 9.8	632.3 ± 10.9	0.4053
	Heart Rate under anesthesia (bpm)	489.9 ± 16.2	417.4 ± 12.2	**0.0071**
	Flow rate aortic arch (mL/min)	22.97 ± 1.819	35.34 ± 3.546	**0.0057**
	Cardiac Output (mL/min)	12.92 ± 0.923	18.42 ± 1.099	**0.0172**
	Stroke Volume (µL)	32.71 ± 1.805	41.30 ± 1.988	0.0503
	Ejection Fraction (%)	50.64 ± 1.989	45.06 ± 2.362	0.1128
	Fraction Area Change (%)	47.08 ± 2.101	38.42 ± 3.723	**0.035**
	Fractional Shortening (%)	14.54 ± 2.094	21.89 ± 1.597	**0.033**
	Left ventricle end-diastolic volume (µL)	65.27 ± 2.186	75.87 ± 4.309	**0.0433**
	Left ventricle end-systolic volume (µL)	31.44 ± 1.005	39.51 ± 3.338	**0.0288**
	Left ventricle mass (mg)	99.09 ± 5.759	145.4 ± 8.825	**0.0039**

By assessing vascular reactivity (Figure 1E), we examined the muscular status of the aorta and found that age limited the vasodilatory response to acetylcholine, while aortas from both groups responded similarly to phenylephrine stimulation. The reduced aortic relaxation mechanism could be attributed to decreased endothelial nitric oxide synthase (eNOS) gene expression (Figure 1C), with inducible nitric oxide synthase (iNOS) seemingly unaffected. The phosphorylation level of eNOS (Figure 1F, 1G) and plasma nitric oxide (NO) levels (Figure 1H) also decreased in the aorta of aged animals.

Beyond cells, the extracellular structures, such as elastic lamellae, are other components that could be affected. AFM (atomic force microscopy) results (Figure 1I, 1J) demonstrated an increase in the Young’s modulus of elastic lamellae in young mice or in the interfiber spaces of aged aortas, consistent with stiffening of elastic fibers and possibly abnormal smooth muscle cell contraction.

### General approaches to tissue structures

**Figure 2:**
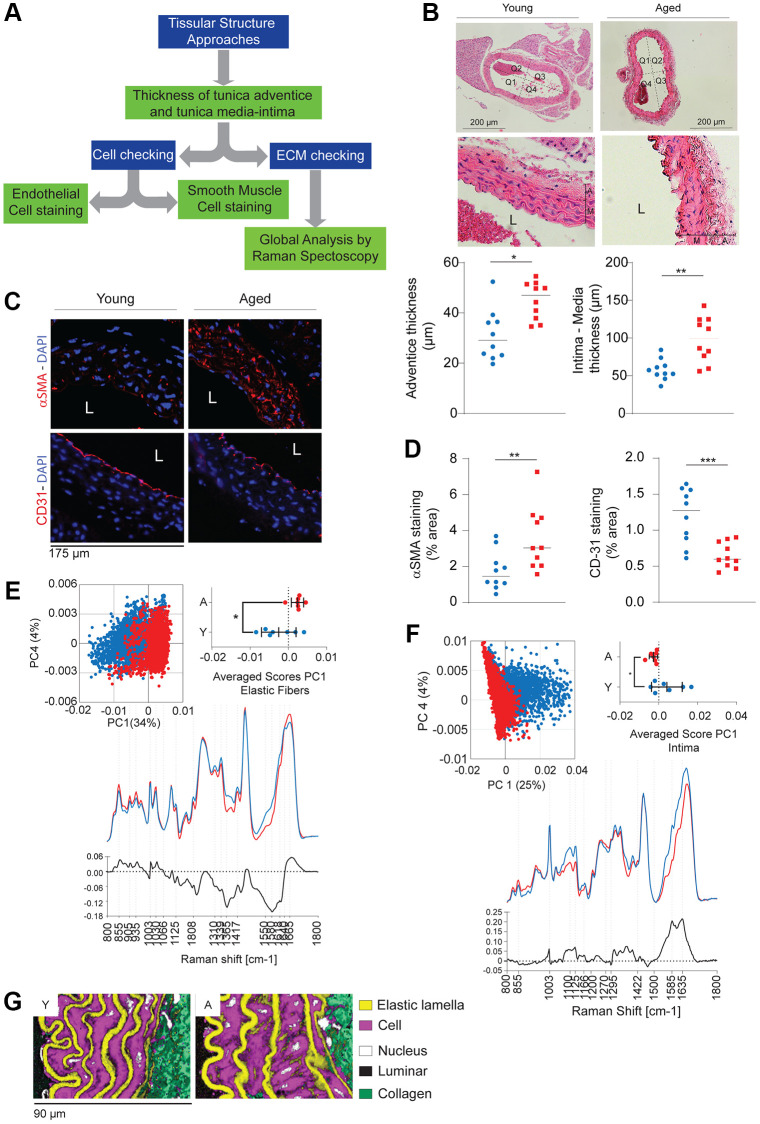
**General evaluation of aortic wall remodeling between old (*n* = 5–10, red) and young (*n* = 5–10, blue) mice.** (**A**) Decision tree allowing cellular and extracellular evaluation. (**B**) Histological staining (Hematoxylin-Eosin, HE) and quantification of the thickness of the tunica media and adventitia by ImageJ Software. (**C**) Immunohistology against αSMA (smooth muscle actin, specific marker for smooth muscle cells) and CD31 (specific marker for endothelial cells). DAPI (blue) identifies cell nuclei. (**D**) Quantification of CD31 and αSMA fluorescence (see Figure 2C) and related to DAPI fluorescence using ImageJ. (**E**, **F**) Examples of the spectra of the elastic fibers (**E**) and the tunica intima (**F**) obtained by Raman spectroscopy. (**G**) Reconstruction of the vascular wall from the spectra obtained previously (panels **E**, **F**). Statistical test: Mann-Whitney. Mean+/− SEM. Significant differences (^*^p < 0.05, ^**^p < 0.001, ^***^p < 0.0001, Mann-Whitney).

Alterations in each of the aortic layers (intima, media, adventitia), changes in cell composition (e.g., SMCs or ECs), and modifications in matrix structures (collagen or elastic fibers) contribute to the reduction in distensibility and/or vascular tone. The thickness of cell layers can be assessed through histological methods (e.g., hematoxylin-eosin - H&E) or immunohistochemistry staining, and by Raman imaging (Figure 2A).

In a morphological analysis of the aortic wall in old and young mice, H&E staining revealed a thicker intima-media and adventitia in old mice compared to young mice (Figure 2B). The increase in media thickness can be partly attributed to the accumulation of SMCs, as indicated by α-SMA immunostaining (Figure 2C, 2D). Discontinuities in the CD31 labeling suggest a loss of ECs in the intimal layer.

Raman spectroscopy, serving as a valuable alternative to histology and immunohistochemistry, excites the molecules within the aortic section under study and detects the diffusion of Raman photons generating a characteristic spectrum (Figure 2E, 2F). Furthermore, it allows for the reconstruction of images from all the collected spectra (Figure 2G). In the case of aging aortas, we observe an accumulation of cells (likely SMCs) in the media and thicker elastic lamellae, suggesting a significant alteration in the ECM.

### Adventitia tunica analysis, focusing on collagen fibers

**Figure 3:**
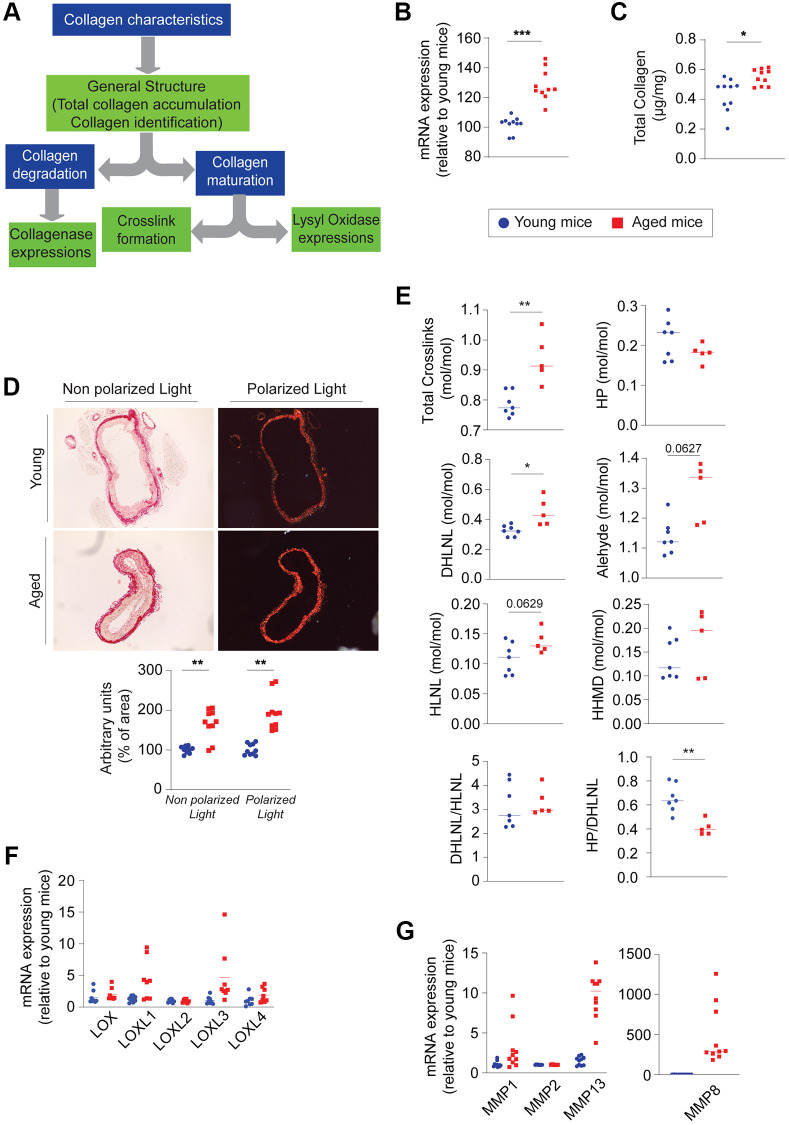
**Evaluation of the collagen component within the aging aortic wall (*n* = 5–10, red) and young (*n* = 5–10, blue).** (**A**) Decision tree allowing the evaluation of collagen. (**B**) mRNA expression of collagen (1a) type I. (**C**) Quantification of total collagen on the whole aorta. (**D**) Histological staining with picrosirius red and visualization in polarized or non-polarized light (left panel). Quantification of colorized collagen as a function of image area using ImageJ Software (right panel). (**E**) Quantification of the different crosslinks formed within collagen fibers such as hydroxylysyl pyridinoline (HP), hydroxylysinonorleucine (HLNL), dehydroxylysinonorleucine (DHLNL), histidinohydroxymerodesmosine (HHMD) in the thoracic aorta. (**F**) mRNA expression of lysyl-oxidase (LOX) and lysyl-oxidase like 1 to 4 (LOXL1, −2, −3, −4). (**G**) mRNA expression of metalloproteinases 1, 2, 13 and 8 (MMP1, −2, −13, −8). Statistical test: Mann-Whitney. Mean +/− SEM. Significant differences (^*^p < 0.05, ^**^p < 0.001, ^***^p < 0.0001, Mann-Whitney).

ECM remodeling in the adventitia primarily involves the deposition of collagen fibers, which offer protection to the aortic wall against overdistension during systolic cardiac phases. Any modification in this structure could either facilitate vessel rupture or, conversely, lead to increased wall rigidity. It’s crucial to analyze the structures of collagen fibers, including assessing their quantity, types, and maturity levels (measured by crosslinking quantification) (Figure 3A). In aged mice, we observed an increase in collagen deposits, particularly type I fibrillar collagen, as indicated by mRNA expression (Figure 3B) and total collagen levels (Figure 3C) in the thoracic aorta. This increase was also confirmed by staining with picrosirius red (PSR) (Figure 3D).

#### Characterization of the maturation-degradation balance of collagen

The birefringence of collagens observed through polarized light microscopy of PSR-stained aorta slices also suggests an increase in type III collagen. The maturation of collagen fibers can be assessed by crosslinking quantification. In the aged thoracic aorta, compared to the young counterpart (Figure 3E), a significant increase is observed in the immature crosslinks, hydroxylysinonorleucine (HLNL), and histidinohydroxymerodesmosine (HHMD), while there’s a decrease in the crosslink of mature hydroxylysine pyridinoline (HP). The immature dehydrohydroxylysinonorleucine (DHLNL) bond remains unchanged. Consequently, the sum of crosslinks (Total XL) increases in older abdominal aortas. The difference observed in the abdominal aorta is less pronounced than in the thoracic aorta (Supplementary Figure 1).

Characteristics of collagen degradation: Assessing the expression of enzymes involved in crosslink development, such as lysyl oxidase (LOX) and lysyl oxidase-like (LOXL-1 to -4), reveals that only LOXL1 and LOXL3 have increased expression in the aged aortas (Figure 3F). Lastly, the expression of collagenases (MMP1, MMP13, and MMP8) is significantly elevated in the old group compared to the young group (Figure 3G). The increase in collagen content and the number of crosslinks suggest that the adventitia layer may stiffen with age.

### Intima-media analysis with a focus on elastic fibers

**Figure 4:**
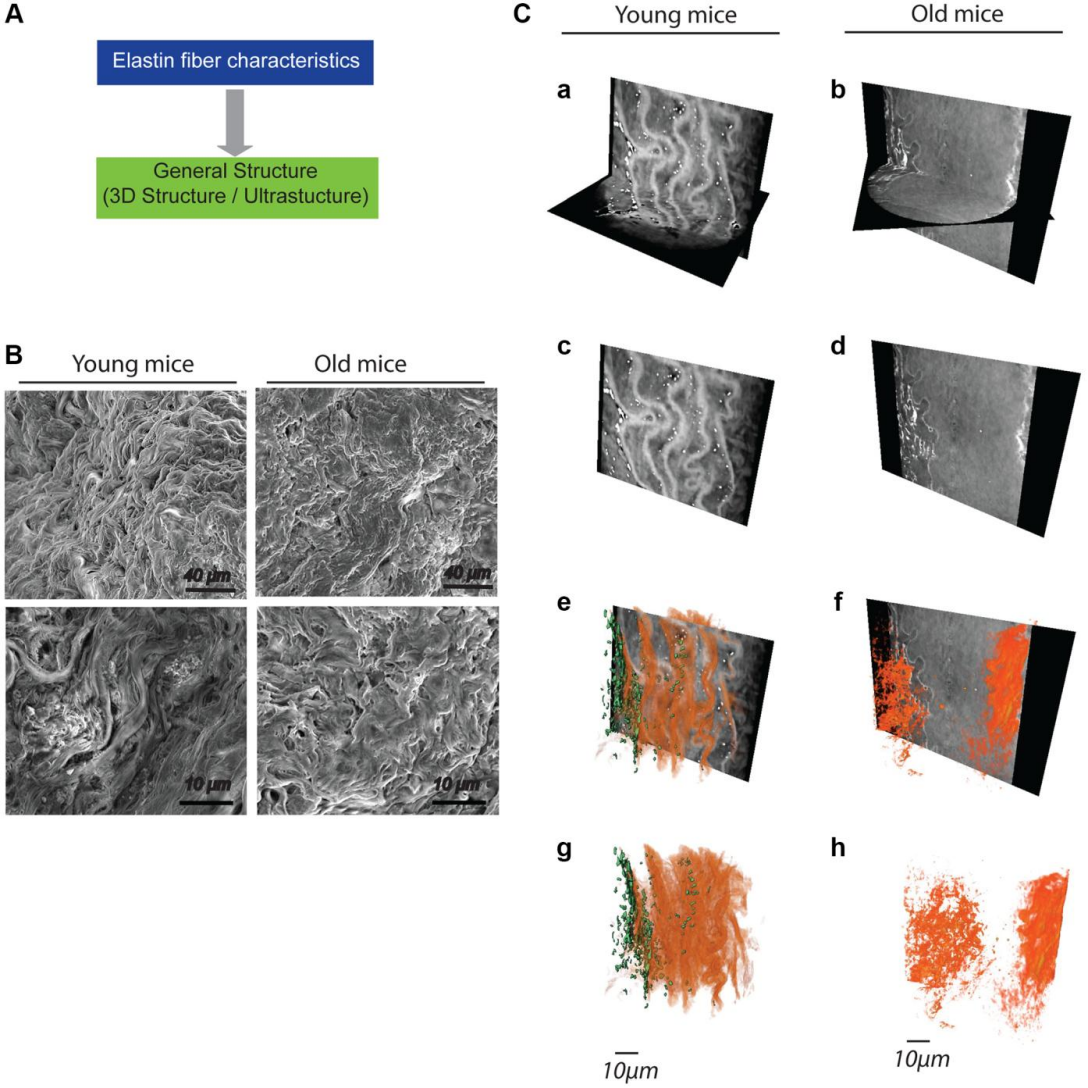
**General impact of aging on the general structuring of elastic fibers within the aortas (*n* = 4/group).** (**A**) Decision tree allowing the evaluation of the general organization of elastic fibers. (**B**) Low (top panels) and high (bottom panels) magnification images obtained from scanning electron microscopy. (**C**) Images obtained by high-resolution X-ray microscopy (panels **a**–**f**). A 3D reconstruction of the network of elastic fibers (red) and cells (green) is made from the images (panels **e**–**h**).

The ECM in the media layer of the aorta is mainly composed of lamellar units of concentric elastic fibers. A thorough examination of this intricate 3D scaffold requires high-resolution microscopy techniques such as scanning electron microscopy (SEM) and 3D X-ray computed tomography (X-ray CT) (Figure 4A). SEM reveals alterations in elastic fibers in aged mice, where the fibers are less distinct compared to those in young aortas (Figure 4B). This disorganization is also visible through X-ray CT (Figure 4C). Simple image segmentation using a grayscale threshold allows for the separation of media cells (pseudo-stained in green) from the elastic lamellae (pseudo-stained in orange) in the young mouse specimen. In the imaged area, the elastic fiber network in the old mouse aorta appears to have a less ordered structure (Figure 4C). Comparing the high-resolution X-ray image of the aorta in an older animal to a younger one, a clear distinction in elastin organization is evident, characterized by a decrease in the quantity and thickness of lamellae, as shown in Figure 4C.

#### Characterization of the elastogenesis

**Figure 5:**
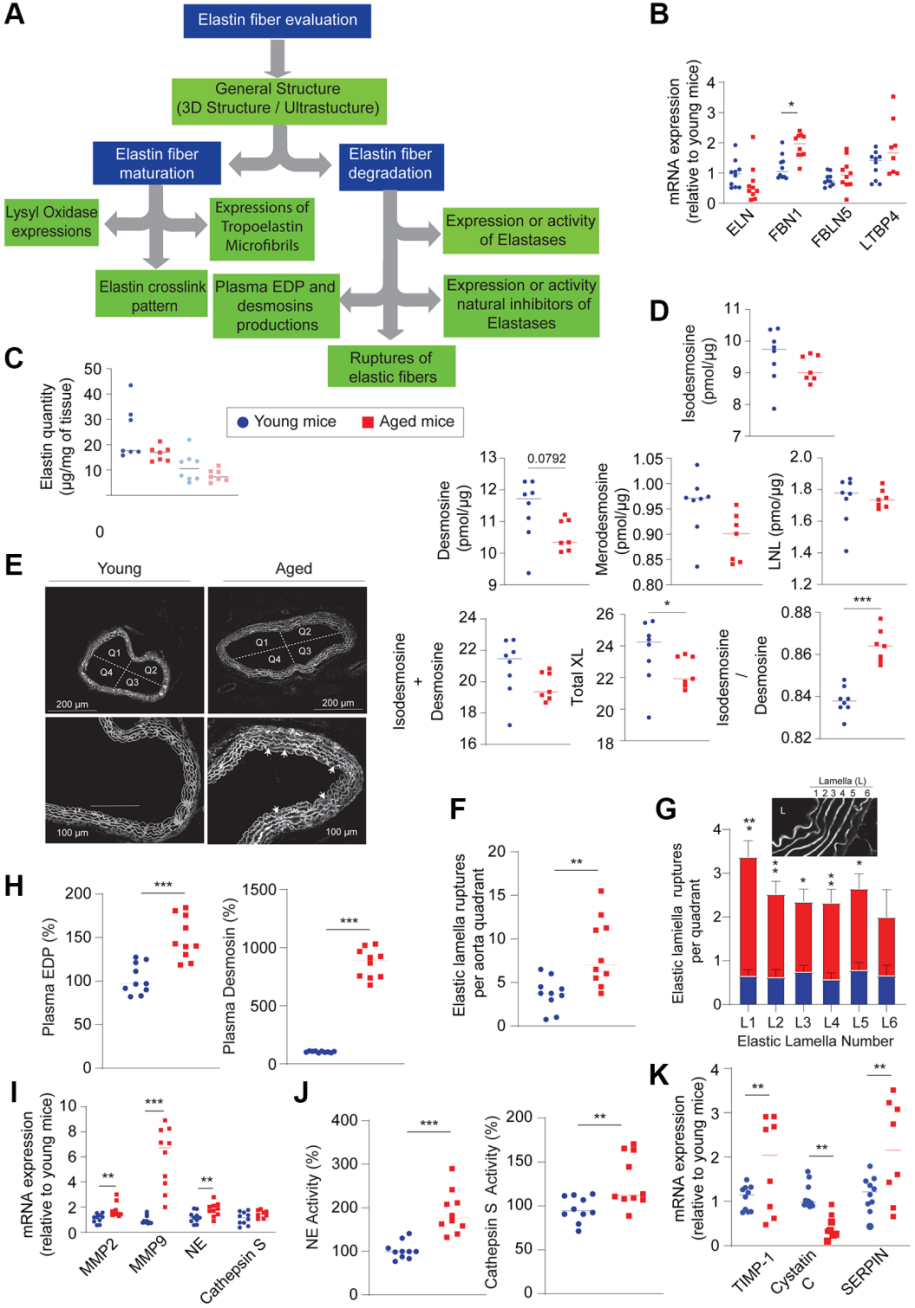
**Evaluation of elastic fibers within the aortic wall (*n* = 5–10, red) and young (*n* = 5–10, blue).** (**A**) Decision tree allowing the evaluation of elastogenesis and elastolysis. (**B**) mRNA expression of elastin (ELN), fibulin 5 (FBLN5), fibrillin 1 (FBN1), latent transforming growth factor beta binding protein 4 (LTBP4). (**C**) Quantification of total elastin on the thoracic aorta (dark blue circle and dark red square) and the abdominal aorta (light blue circle and light red square). (**D**) Quantification of the different crosslinks formed within elastic fibers such as isodesmosine, desmosine, merodesmosine, lysinonorleucine (LNL) in the thoracic aorta. (**E**) Visualization of the ruptures (arrowheads) of the elastic lamellae observed by the autofluorescence of elastin. (**F**) Total quantification of the ruptures observed in Figure 5E. (**G**) Counting the number of ruptures observed for each elastic lamella and determining from Figure E. (**H**) Plasma quantification of elastin-derived peptides (EDP) and desmosines. (**I**) mRNA expression of elastases such as matrix metalloproteinase 2 and 9 (MMP2 and MMP9), neutrophil elastase (NE) and cathepsin S. (**J**) Plasma activities of neutrophil elastase (NE) and cathepsin S. (**K**) mRNA expression of natural elastase inhibitors, TIMP1 (tissue inhibitor of metalloproteinase), cystatin C, Serine protein inhibitor (SERPIN). Statistical test: Mann-Whitney. Mean +/− SEM. Significant differences (^*^p < 0.05, ^**^p < 0.001, ^***^p < 0.0001, Mann-Whitney).

The observed changes in the elastic fiber scaffold in the aged mouse specimen are likely due to elastolysis processes, including elastin fragmentation, elastin peptide production, and elastase activities. Nevertheless, elastogenesis and elastosis should not be overlooked (Figure 5A). Elastogenesis is characterized by the expression of elastin (ELN) and microfibrillar glycoproteins, such as fibrillins (e.g., FBN1), fibulins (e.g., FBLN5), or latent transforming growth factor-beta binding proteins (e.g., LTBP4), and the formation of crosslink patterns that indicate fiber maturity.

When comparing aged and young mouse aortas, the expression of mRNAs for ELN, FBLN-5, and LTBP4 is identical in both groups studied (Figure 5B). Only the mRNA expression of FBN-1 is significantly increased. Furthermore, the total insoluble elastin decreases, suggesting a loss of integrity of this protein (Figure 5C). When evaluated individually, no significant difference is observed in crosslinks within the thoracic (or abdominal) aorta of the two groups (Figure 5D and Supplementary Figure 1). However, the sum of all different crosslinking patterns (Total XL) significantly decreases in aged aortas compared to young aortas, confirming the disorganization of these fibers.

#### Characterization of the elastolysis

In addition to assessing elastogenesis, understanding elastolysis is a crucial aspect for determining the degree of vascular stiffness (Figure 5A). Histological methods such as Hart’s staining or elastin autofluorescence enable an estimation of the integrity of elastic fibers by measuring their thickness or identifying ruptures. To facilitate measurements, the vessel is divided into four quadrants (Figure 5E). The total number of elastic fiber ruptures is higher in old aortas compared to young aortas (Figure 5F). Examining individual fibers, we observe these ruptures across all six concentric lamellae without distinction (Figure 5G). Elastic fiber fragmentation can also be determined by measuring the quantity of elastin-derived peptides (EDP) or desmosines found in the plasma, which are significantly increased in aged mice (Figure 5H).

Another approach to detect increased elastolysis within aged aortas is quantifying the expression of elastases such as MMP2, MMP9, neutrophil elastase, and cathepsin S (Figure 5I), or the activity of cathepsin S in plasma (Figure 5J). However, these increased activities can be counterbalanced by the presence of their natural inhibitors. In older mice, inhibitors of MMP9, neutrophil elastase, or cathepsin S (e.g., TIMP1 (tissue inhibitor of metalloproteinase), SERPIN (Serine protein inhibitor), cystatin C, respectively) have significantly increased expression (Figure 5K), suggesting a mitigation of the effects of proteases.

## DISCUSSION

Among the critical factors contributing to vascular aging, the deformability of elastic arteries, as determined by elastance-compliance, plays a significant role, possibly on par with or even surpassing the impact of hypertension [13, 14]. In this study, we have, for the first time, amalgamated classical and innovative methods into a single framework for assessing vascular stiffness, considering both cellular and extracellular parameters that may influence it. We have given precedence to methodologies both in terms of function and anatomy (see Figure 1A and Supplementary Figure 2) to streamline future research, be it fundamental, clinical, or diagnostic, in the field of vascular stiffness.

The points we address in these decision trees allow for a comprehensive characterization of vascular stiffness for preclinical studies. It is important to identify the cellular component (i.e., endothelial and smooth muscle cells), as numerous studies have already illustrated. In these same studies, the contribution of extracellular matrix remodeling is generally not mentioned. This could be due to the authors’ lack of knowledge and/or experience in ECM analysis.

However, whether for preclinical or clinical studies, it is crucial to identify the reasons for vascular stiffness —cells or ECM— as therapeutic approaches can be very different.

In clinical conditions, few parameters are currently available using non-invasive imaging methods, such as PWV and aortic elasticity (Figure 1A and Supplementary Table 1). Plasma biomarkers can also be evaluated: levels of NO, endothelin, elastase activity, or soluble elastin fragments (Supplementary Figure 2 and Supplementary Table 1).

Although the structures of mouse and human aortas are quite different, the aging process between young and old mice compared to humans is remarkably comparable (Supplementary Table 1). This comparison underscores the relevance of our methods across species. To illustrate the practicality of this methodological approach, we applied our decision trees to a study comparing aortic stiffness in old and young mice (without apparent pathology).

In our proposed decision trees, the primary factor under scrutiny by clinicians and basic researchers is the functional alteration of SMCs and/or ECs. These disruptions often lead to increased SMC contraction due to elevated levels of plasma vasoconstrictor agents, such as endothelin 1, and a reduction in pro-relaxing factors like nitric oxide production. Furthermore, SMC hyperproliferation intensifies vasoconstriction. When evaluating vascular stiffness and its impact on systolic pressure, the remodeling of cellular components, including SMCs and ECs, should not be overlooked. It is important to note that functional measurements, except for blood pressure, were performed in anesthetized mice. Anesthesia can undoubtedly minimize the differences between groups, here elderly vs. young individuals. The application of these methods in awake individuals, as in humans, would undoubtedly be more relevant. Indeed, these functional measurements can be performed on awake patients, either in specialized medical imaging facilities or by a general practitioner trained in ultrasound and pulse wave velocity measurement.

Although the cellular component significantly contributes to vascular resistance, the fundamental elements of the ECM (elastic and collagen fibers) play a crucial role in maintaining aortic structural and functional integrity. Therefore, during systolic phases, collagen fibers bear more than half of the mechanical load on the vessel, serving to safeguard the aortic wall from overdistension. When collagen synthesis is downregulated or when structural alterations occur, the vessel wall becomes more susceptible to fatigue and failure, increasing the likelihood of multiple arterial ruptures and aortic dissections [15, 16]. On the contrary, an excess of these fibers or excessive cross-linking between them has been associated with elevated arterial pressures and vascular stiffening [17–19].

In addition to these structural components, the inflammatory state of the arterial wall also plays a critical role in vascular aging. Chronic, low-grade inflammation is a common feature of aging tissues and contributes significantly to vascular dysfunction. During aging, low-grade inflammation is consistently observed in many tissues. This is particularly evident in the arterial component, where immune cell infiltration is noted. Such inflammation, which could be quantified by flow cytometry, can lead to cellular dysfunction by disrupting molecular processes like autophagy, proliferation, differentiation, and cellular calcification. Additionally, the presence of leukocytes can release proteases (such as neutrophil elastase, cathepsin S, and MMPs), contributing to the degradation of both collagen and elastic fibers. The degradation of these fibers due to inflammatory processes further complicates the structural integrity of the aorta. This interaction between inflammation and ECM remodeling underscores the need for comprehensive visualization techniques to assess fiber integrity.

Collagen visualization can be accomplished using various classic, rapid, and cost-effective histological methods, such as Masson’s trichrome and picrosirius red (PSR). Unlike Masson’s trichrome, PSR allows for the visualization of both collagen fiber types I and III [20]. When observed under polarized light microscopy, PSR-stained samples exhibit type I collagen fibers (thicker fibers) with yellow-orange birefringence and type III collagen (thinner fibers) with green birefringence. However, it’s worth noting that some studies have contested this differentiation. In our study, we have adopted a comprehensive quantification approach that encompasses both type I and type III collagen fibers.

Alternatively, a more costly solution involves multiphoton microscopy with second harmonic generation (SHG), where the primary source of the signal is fibrillar collagen (type I). This technique allows for the identification of the macromolecular organization of fibrillar collagen (type I) exclusively, and it permits the application of specific immunostaining for other collagen types. Significantly increased expression of aortic collagen types I and III, during aging, may result in impaired fibril formation and, consequently, decreased tensile strength of vascular tissue [21, 22]. These increases, particularly in type III collagen, could be a warning sign of a risk of dissection and aneurysm.

While the accumulation of collagen fibers affects the radius-tension relationship (Laplace’s law), the elastic lamellae confer elastance and compliance properties to blood vessels. Several methods are available to study the degree of elasticity (or stiffening) in elastic fibers. Ultrasound and MRI approaches allow us to determine the degree of aorta stiffening by measuring Young’s modulus, compliance, or pulse-wave velocity. These techniques are readily accessible in healthcare institutions, making it reasonable to consider them for diagnosing vascular rigidity. We directly determined the Young’s modulus of elastic fibers, as described by Berquant et al. [23]. Young’s modulus measurements obtained through ultrasound approaches (*in vivo*) or AFM (*in vitro*) reveal a significant stiffening of elastic fibers in aged aortas compared to those in young mice, suggesting a modification in the integrity of these elastic fibers.

Elastic lamellae, or elastic fibers, possess a complex structure comprising numerous proteins. The most abundant among these is a large biopolymer known as elastin, which is stabilized by microfibrils and covalent cross-links. These microfibrils include fibrillins, fibulins, and latent transforming growth factor-β binding proteins (LTBPs). Simultaneously, cross-linking enzymes such as lysyl oxidase (LOX) and lysyl oxidase-like 1 facilitate intra-fiber crosslinks, including desmosine, isodesmosine, or merodesmosine, markers of elastic fibers maturity. Current methods for identifying the development of the scaffold forming the elastic fiber are mostly limited to mRNA or protein expression, providing information only regarding the presence or absence of elastin and microfibrils. In contrast, biochemical analysis of crosslinks offers insight into fiber maturity.

Combining various imaging methods such as Raman spectroscopy, three-dimensional X-ray imaging, and scanning electron microscopy can complement biochemical approaches. Images obtained by high-resolution X-ray imaging (Figure 4C) show a morphological change likely due to lamellae rupture in old animals. The visualization of lamellae differs between SEM and X-ray imaging, with SEM capturing surface details while high-resolution X-ray imaging provides insight into the internal structure. This discrepancy highlights the value of using multiple microscopic methods to understand elastin distribution in biological samples. Raman spectroscopy and X-ray tomography do not require molecular labeling or contrast agents and yield high-resolution quantitative images of the spatial distribution of ECM constituents in biological tissue sections (paraffin or frozen), living tissue, or even *in vivo* (e.g., fiber optics coupled with Raman spectroscopy) [24]. This minimally invasive method, although still experimental, holds potential for aiding in the pathological diagnosis of ECM [25]. In the absence of techniques like Raman spectroscopy, standard histological methods (e.g., Weigert’s, van Gieson’s, Hart’s staining) [11], or elastin autofluorescence [20], provide information about the general appearance of the fibers (thickness, quantity) and are user-friendly.

Several studies, including our own, reveal that three-dimensional X-ray imaging and scanning electron microscopy are currently the only approaches that offer high-resolution images to visualize ruptures and defects in the fiber architecture of the intima-media tunica. Fractures can be identified [26, 27], as well as the interaction of the elastic laminae with other surrounding tissues (e.g., perivascular adipose tissue) [28, 29]. However, these methods cannot provide information about the elastic properties of the fibers during systole or diastole, necessitating simulations of these cardiovascular phases by pressurizing the vessel upstream of the imagery [30].

Nonetheless, these imaging methods are primarily limited to providing morphological information and lack the resolution to detect local molecular events on elastic fibers or collagen, such as post-translational modifications (e.g., glycation, carbamylation, N-homocysteinylation, glycosylation, etc.) or crosslinks between elastin fibers (desmosine, isodesmosine), indicative of fiber aging [31, 32]. Expression levels (mRNA or proteins) and cross-links can be measured relatively easily [20, 33, 34]. In our study involving old mice, we observe that collagen cross-links are favored, while those of elastic fibers are reduced compared to young mice. This suggests a loss of structural integrity in elastic fibers in favor of collagen fibers, contributing to vessel stiffening. These alterations in elastic fibers, including fragmentations evident through elastin autofluorescence or reduced cross-link quantities, can be detected through measurements of desmosines and/or elastin-derived peptides (soluble fraction of elastin) in plasma [20, 35]. These markers correlate with aging [36, 37]. In keeping with Paczek et al., we demonstrate that the synthesis and plasma activity of proteases (e.g., cathepsin S, neutrophil elastase) increase with age, potentially explaining age-related diseases and arterial stiffening [38, 39]. Consequently, these plasma assays, including these protease activities or elastin fragments degraded (EDP and/or desmosines), could easily be performed by medical analytical laboratories. Those plasma data coupled with functional imagery (MRI or ultrasound) could integrate into clinical diagnostic methods and provide the general practitioner or specialist with major information for diagnosing arterial stiffening. Then, the complications of vascular stiffness could be anticipated and a therapeutic strategy adapted, such as an anti-hypertensive treatment, for example.

As a proof of concept for future studies in humans, it would be interesting to use our functional and anatomical decision trees to provide a risk score that could predict the occurrence of future arterial stiffness and hypertension. This risk score should take into account functional data such as pulse wave velocity (>10 m/s) and/or Young’s modulus (<1.8 × 10^3^ kPa) of the aorta obtained by ultrasound or MRI [40, 41]. Additionally, this score would be augmented by plasma factors such as endothelial markers (NO, <22 mM and ET-1, >2.02 pg/mL) [42, 43] and factors involved in elastolysis, such as plasma activities of elastases (NE, >243.9 ng/mL and cathepsin S, >26.28 ng/mL) [44, 45], as well as markers of elastic fiber degradation (i.e., elastin-derived peptides (EDP, >4.9 ng/mL and/or plasma desmosines, >0.4 ng/mL) [46–48]. The resulting score could allow clinicians to anticipate the implementation of antihypertensive treatments, for example. Moreover, all these data could eventually be integrated into other cardiovascular risk scores, such as the Framingham Risk Score. This score predicts various aspects of cardiovascular disease risk by integrating the age and gender of the patient, their systolic blood pressure, total plasma cholesterol levels, HDL, and CRP. It also considers whether patients are diabetic, smokers, and the treatments they use. But this Framingham Risk Score, as a lot of other cardiovascular scoring systems, can explain no more than 50% of the overall risk of developing cardiovascular disease [49–52]. Improving the robustness of these scores could involve integrating parameters describing vascular stiffness in our study into the calculation method.

The study concludes by emphasizing the need for a multimodal and multi-level approach to study arterial stiffening, providing valuable insights for diagnosis and preclinical research into molecular mechanisms. Detecting and preventing aortic stiffness is deemed crucial in clinical practice and fundamental research, suggesting the integration of new diagnostic options into routine medical practices. However, the article acknowledges the current applicability of non-invasive imaging approaches primarily in preclinical research and calls for the development of low-cost, easy, and rapid protocols for clinical use.

## MATERIALS AND METHODS

### Animal models

All mouse procedures conformed to the Guide for Care and Use of Laboratory Animals of the US National Institutes of Health and were approved by the Animal Subjects Committee of Champagne-Ardenne (2018032113171684v8-15413). C57BL/6N mice (6 and 20 months old) were purchased from Janvier (Le Genest-Saint-Isle, France) and housed in a 12:12-hour light/dark cycle, temperature- and humidity-controlled environment. They had ad libitum access to a standard diet (AIN-93 M rodent diet, Special Diet Service, UK), and water during the experimental period.

### Functional parameters

#### Measurement of blood pressure

The study employs the non-invasive tail-cuff method to measure blood pressure in animals, avoiding anesthesia or surgery. Adaptation and handling are required, with regular daily sessions. Parameters include pulse, systolic and diastolic pressures, from which mean arterial pressure and pulse pressure are derived. Hypertension is determined based on statistical differences in pressure between aged and young mice.

#### Pulse wave velocity (PWV)

Velocity measurements are conducted in anesthetized animals (4% isoflurane, 10 min). The measurement can be performed using ultrasound probes (Indus Instruments, Webster, TX, USA), with one probe placed at the level of the aortic arch and the other at the level of the abdominal thoracic aorta near the bifurcation of the iliac arteries. While Doppler ultrasound systems can measure velocity limited to specific portions of arteries (such as carotids and the aortic arch), this method considers the entire aorta, providing a more comprehensive assessment of vessel rigidity.

#### High-frequency ultrasound imaging

Under isoflurane anesthesia, we measured heart parameters. The Vevo3000 system assessed left ventricle functions, aortic arch dimensions, pulse wave velocity, and intima-media thickness. Distensibility factor (D) and Young’s modulus (E) were derived using relevant equations such as: the Bramwell and Hill equation [53] and the Moens-Korteweg equation. Pressure variation and compliance were deduced following Brands’ methodology [54].

#### Vascular reactivity

We investigated the effects of aging on *ex vivo* aortic reactivity. Wild-type mice were anesthetized by intraperitoneal injections of pentobarbital sodium (150 mg/kg). Once euthanasia was complete, their hearts were removed. Vascular reactivity studies were carried out, as described in [55].

### Biochemical parameters

#### Plasma assay

Peripheral blood from mice was collected in heparinized tubes by retroorbital puncture, and the plasma was stored at −80°C. Evaluations of elastin-derived peptide concentrations were performed using a commercially available kit (Biocolor, Antrim, UK) according to [56] and a desmosine ELISA kit (Cusabio Technology LLC, Houston, TX, USA) according to [20]. A neutrophil elastase activity assay and a cathepsin activity assay (Abcam, Cambridge, UK) were used to measure neutrophil elastase and cathepsin S activities, respectively [20]. Endothelin1 was quantified with an ELISA kit (Mybiosource, Vancouver, Canada).

#### Total collagen and elastin quantifications

Tissue was digested with HCl and its collagen content was determined from collagen assay kit (Sigma-Aldrich, St. Louis, MO, USA). Quantification of total elastin was carried out according to the protocol described in the study by Nave et al. [57].

#### Cross-linking assay

Protein analysis followed the protocol described [57]. Collagen crosslink analysis involved sodium borohydride reduction, bacterial collagenase digestion, and HCl hydrolysis. Crosslinked soluble fractions were hydrolyzed and precleared, with eluates analyzed on an amino acid analyzer. Crosslink nomenclature denotes reduced variants. Collagen content, based on hydroxyproline, was calculated from hydrolyzed collagenase-soluble samples. For protein and elastin crosslinks, samples underwent collagenase digestion. Soluble collagen underwent hydrolysis, while the residual fraction was alkali-extracted. Elastin and non-collagenous proteins in the supernatant were hydrolyzed. Elastin crosslinks in the NaOH-insoluble fraction were analyzed after CF-11 preclearance. The study’s approach provides insights into collagen, protein, and elastin crosslinks.

#### Gene expression

The analysis utilized qPCR, with RNA extraction, cDNA synthesis, and real-time PCR as previously described [20]. The housekeeping genes 36B4 and RPS26 normalized RNA expression, calculated using the 2−ΔΔCT method. Detailed primer sequences are available in Supplementary Table 2. The study’s comprehensive approach provides insights into collagen, protein, elastin crosslinks, and gene expression.

#### Western blotting

Western blotting was performed as previously described by Blaise et al. [56]. The antibodies, including endothelial nitric oxide synthase (eNOS, 32027S, dilution 1/1000) and its phosphorylated form (p-NOS, 9570S, dilution 1/1000), were purchased from Cell Signaling Technology (Danvers, MA, USA).

### Imaging parameters

#### Histology approaches

From paraffin cross-sections (5 µm thickness) of aortas, deparaffinization and rehydration were performed before staining with Hematoxylin-Eosin (H&E) or red picrosirius, as described [20]. Autofluorescence of elastin at a wavelength of 488 nm was observed from the H&E staining. Thickness measurements of intima-media and adventitia tunica were conducted using the ImageJ Software.

#### Raman spectroscopy/imaging

Raman measurements used a Witec alpha 300R confocal Raman microscope on deparaffinized aorta cross-sections. Samples were kept hydrated, and underwent imaging with a green laser (532 nm). Two images per sample were acquired, each covering an 80 × 90 µm area at 0.5 × 0.5 µm/pixel resolution. Data preprocessing included cosmic ray removal, baseline correction, cropping, and normalization. True component analysis (TCA) generated intensity distribution heatmaps, identifying prevalent spectral signatures. Principal component analysis (PCA) on single spectra extracted from TCA images allowed in-depth molecular analysis. PCA was applied separately for elastic fibers and interfibrillar ECM.

#### Atomic force microscopy (AFM)

Frozen 10 µm-thick aorta cross-sections were incubated in KH solution for equilibration at 37°C. The prepared samples were placed onto the microscope stage and observed with bright field illumination to locate the spots of interest. Analysis was performed using AFM as described [23, 58]. Analyses were performed at three different locations in each cross-section, for a total of nine cross-sections obtained from three different mice.

#### High-resolution X-ray microscopy

Aorta underwent paraffin removal and staining before manual sectioning. Sections were affixed to metallic pins for X-ray imaging using a Carl Zeiss Xradia 810 Ultra microscope with a chromium source. Zernike phase-contrast and a field-of-view of 64 µm² were employed for imaging. A total of 901 projection images were acquired during a 180-degree sample rotation. Reconstruction, using a filtered back-projection algorithm, resulted in isotropic voxel-sized (128 nm) volumetric images. Stitching of images was performed, and tomograms were exported for visualization in Thermo Fischer Avizo software. This methodology provided detailed X-ray images for comparative analysis of aortic structures in young and old mice.

#### Scanning electron microscopy

Defrosted samples were deposited onto a SEM stub and treated with NanoSuit^®^ Aqueous Solution (Electron Microscopy Sciences) according to the manufacturer’s instructions. Samples were imaged in a Scanning Electron Microscope FEI Quanta 3D FEG Dual-Beam working at an acceleration voltage of 5 kV.

### Statistical analysis

Data were prospectively collected and analyzed using StatView 5 software for Windows (SAS Institute, Berkley, CA, USA). In agreement with random sampling analyses, comparisons between groups are presented as mean ± SEM. Nonparametric statistics (Mann-Whitney *U*-test) were used. For all analyses, a *p*-value < 0.05 was considered to indicate statistical significance.

### Availability of data and materials

The authors declare that all supporting data are available within the article and its Supplementary Materials. The corresponding authors may provide additional data supporting the findings of this study upon reasonable request.

## Supplementary Materials

Supplementary Materials

Supplementary Figures

Supplementary Tables

## References

[1] NCD Risk Factor Collaboration (NCD-RisC). Worldwide trends in hypertension prevalence and progress in treatment and control from 1990 to 2019: a pooled analysis of 1201 population-representative studies with 104 million participants. Lancet. 2021; 398:957–80. 10.1016/S0140-6736(21)01330-1 34450083 PMC8446938

[2] Zhang Y, Lacolley P, Protogerou AD, Safar ME. Arterial Stiffness in Hypertension and Function of Large Arteries. Am J Hypertens. 2020; 33:291–6. 10.1093/ajh/hpz193 32060496

[3] Boutouyrie P. Estimating Is Not Measuring: The Lessons About Estimated Pulse Wave Velocity. J Am Heart Assoc. 2022; 11:e025830. 10.1161/JAHA.122.025830 35535609 PMC9238559

[4] Justin J, Fayol A, Bruno RM, Khettab H, Boutouyrie P. International Guidelines for Hypertension: Resemblance, Divergence and Inconsistencies. J Clin Med. 2022; 11:1975. 10.3390/jcm11071975 35407581 PMC9000018

[5] Duca L, Blaise S, Romier B, Laffargue M, Gayral S, El Btaouri H, Kawecki C, Guillot A, Martiny L, Debelle L, Maurice P. Matrix ageing and vascular impacts: focus on elastin fragmentation. Cardiovasc Res. 2016; 110:298–308. 10.1093/cvr/cvw061 27009176

[6] Prenner SB, Chirinos JA. Arterial stiffness in diabetes mellitus. Atherosclerosis. 2015; 238:370–9. 10.1016/j.atherosclerosis.2014.12.023 25558032

[7] Elias MF, Crichton GE, Dearborn PJ, Robbins MA, Abhayaratna WP. Associations between Type 2 Diabetes Mellitus and Arterial Stiffness: A Prospective Analysis Based on the Maine-Syracuse Study. Pulse (Basel). 2018; 5:88–98. 10.1159/000479560 29761082 PMC5939695

[8] Monteiro CI, Simões RP, Goulart CL, Silva CDD, Borghi-Silva A, Mendes RG. Arterial stiffness in type 2 diabetes: determinants and indication of a discriminative value. Clinics (Sao Paulo). 2021; 76:e2172. 10.6061/clinics/2021/e2172 33624706 PMC7885854

[9] Mitchell GF. Arterial Stiffness in Aging: Does It Have a Place in Clinical Practice?: Recent Advances in Hypertension. Hypertension. 2021; 77:768–80. 10.1161/HYPERTENSIONAHA.120.14515 33517682

[10] Aroor AR, Jia G, Sowers JR. Cellular mechanisms underlying obesity-induced arterial stiffness. Am J Physiol Regul Integr Comp Physiol. 2018; 314:R387–98. 10.1152/ajpregu.00235.2016 29167167 PMC5899249

[11] Wagenseil JE, Mecham RP. Elastin in large artery stiffness and hypertension. J Cardiovasc Transl Res. 2012; 5:264–73. 10.1007/s12265-012-9349-8 22290157 PMC3383658

[12] Jacob MP. Extracellular matrix remodeling and matrix metalloproteinases in the vascular wall during aging and in pathological conditions. Biomed Pharmacother. 2003; 57:195–202. 10.1016/s0753-3322(03)00065-9 12888254

[13] Wagenseil JE, Mecham RP. Vascular extracellular matrix and arterial mechanics. Physiol Rev. 2009; 89:957–89. 10.1152/physrev.00041.2008 19584318 PMC2775470

[14] Pinto E. Blood pressure and ageing. Postgrad Med J. 2007; 83:109–14. 10.1136/pgmj.2006.048371 17308214 PMC2805932

[15] Vouyouka AG, Pfeiffer BJ, Liem TK, Taylor TA, Mudaliar J, Phillips CL. The role of type I collagen in aortic wall strength with a homotrimeric. J Vasc Surg. 2001; 33:1263–70. 10.1067/mva.2001.113579 11389427

[16] Assavarittirong C, Au TY, Nguyen PV, Mostowska A. Vascular Ehlers-Danlos Syndrome: Pathological Variants, Recent Discoveries, and Theoretical Approaches. Cardiol Rev. 2022; 30:308–13. 10.1097/CRD.0000000000000419 34560710

[17] Narayanan N, Pushpakumar SB, Givvimani S, Kundu S, Metreveli N, James D, Bratcher AP, Tyagi SC. Epigenetic regulation of aortic remodeling in hyperhomocysteinemia. FASEB J. 2014; 28:3411–22. 10.1096/fj.14-250183 24739303 PMC4101656

[18] Fu S, Li Y, Wu Y, Yue Y, Yang D. Icariside II improves myocardial fibrosis in spontaneously hypertensive rats by inhibiting collagen synthesis. J Pharm Pharmacol. 2020; 72:227–35. 10.1111/jphp.13190 31820448

[19] Li Y, Tai HC, Sladojevic N, Kim HH, Liao JK. Vascular Stiffening Mediated by Rho-Associated Coiled-Coil Containing Kinase Isoforms. J Am Heart Assoc. 2021; 10:e022568. 10.1161/JAHA.121.022568 34612053 PMC8751888

[20] Romier B, Dray C, Vanalderwiert L, Wahart A, Hocine T, Dortignac A, Garbar C, Garbar C, Boulagnon C, Bouland N, Maurice P, Bennasroune A, Sartelet H, et al. Apelin expression deficiency in mice contributes to vascular stiffening by extracellular matrix remodeling of the aortic wall. Sci Rep. 2021; 11:22278. 10.1038/s41598-021-01735-z 34782679 PMC8593139

[21] Bode MK, Soini Y, Melkko J, Satta J, Risteli L, Risteli J. Increased amount of type III pN-collagen in human abdominal aortic aneurysms: evidence for impaired type III collagen fibrillogenesis. J Vasc Surg. 2000; 32:1201–7. 10.1067/mva.2000.109743 11107093

[22] Wang X, LeMaire SA, Chen L, Shen YH, Gan Y, Bartsch H, Carter SA, Utama B, Ou H, Coselli JS, Wang XL. Increased collagen deposition and elevated expression of connective tissue growth factor in human thoracic aortic dissection. Circulation. 2006; 114:I200–5. 10.1161/CIRCULATIONAHA.105.000240 16820572 PMC2637375

[23] Berquand A, Wahart A, Henry A, Gorisse L, Maurice P, Blaise S, Romier-Crouzet B, Pietrement C, Bennasroune A, Sartelet H, Jaisson S, Gillery P, Martiny L, et al. Revealing the elasticity of an individual aortic fiber during ageing at nanoscale by in situ atomic force microscopy. Nanoscale. 2021; 13:1124–33. 10.1039/d0nr06753a 33399602

[24] Bergholt MS, Albro MB, Stevens MM. Online quantitative monitoring of live cell engineered cartilage growth using diffuse fiber-optic Raman spectroscopy. Biomaterials. 2017; 140:128–37. 10.1016/j.biomaterials.2017.06.015 28649013 PMC5504667

[25] Bergholt MS, Lin K, Wang J, Zheng W, Xu H, Huang Q, Ren JL, Ho KY, Teh M, Srivastava S, Wong B, Yeoh KG, Huang Z. Simultaneous fingerprint and high-wavenumber fiber-optic Raman spectroscopy enhances real-time in vivo diagnosis of adenomatous polyps during colonoscopy. J Biophotonics. 2016; 9:333–42. 10.1002/jbio.201400141 25850576

[26] Heinz A, Huertas AC, Schräder CU, Pankau R, Gosch A, Schmelzer CE. Elastins from patients with Williams-Beuren syndrome and healthy individuals differ on the molecular level. Am J Med Genet A. 2016; 170:1832–42. 10.1002/ajmg.a.37638 27311421

[27] Mora Huertas AC, Schmelzer CE, Hoehenwarter W, Heyroth F, Heinz A. Molecular-level insights into aging processes of skin elastin. Biochimie. 2016; 128-129:163–73. 10.1016/j.biochi.2016.08.010 27569260

[28] Ben Zemzem A, Genevaux A, Wahart A, Bodey AJ, Blaise S, Romier-Crouzet B, Jonquet J, Bour C, Cogranne R, Beauseroy P, Dauchez M, Sherratt MJ, Debelle L, Almagro S. X-ray microtomography reveals a lattice-like network within aortic elastic lamellae. FASEB J. 2021; 35:e21844. 10.1096/fj.202100323RR 34473371 PMC12316089

[29] Ben Zemzem A, Liang X, Vanalderwiert L, Bour C, Romier-Crouzet B, Blaise S, Sherratt MJ, Weitkamp T, Dauchez M, Baud S, Passat N, Debelle L, Almagro S. Early Alterations of Intra-Mural Elastic Lamellae Revealed by Synchrotron X-ray Micro-CT Exploration of Diabetic Aortas. Int J Mol Sci. 2022; 23:3250. 10.3390/ijms23063250 35328674 PMC8954876

[30] Shearer T, Bradley RS, Hidalgo-Bastida LA, Sherratt MJ, Cartmell SH. Three-dimensional visualisation of soft biological structures by X-ray computed micro-tomography. J Cell Sci. 2016; 129:2483–92. 10.1242/jcs.179077 27278017

[31] Wahart A, Hocine T, Albrecht C, Henry A, Sarazin T, Martiny L, El Btaouri H, Maurice P, Bennasroune A, Romier-Crouzet B, Blaise S, Duca L. Role of elastin peptides and elastin receptor complex in metabolic and cardiovascular diseases. FEBS J. 2019; 286:2980–93. 10.1111/febs.14836 30946528

[32] Wagenseil JE, Mecham RP. New insights into elastic fiber assembly. Birth Defects Res C Embryo Today. 2007; 81:229–40. 10.1002/bdrc.20111 18228265

[33] Tsang KM, Knutsen RH, Billington CJ Jr, Lindberg E, Steenbock H, Fu YP, Wardlaw-Pickett A, Liu D, Malide D, Yu ZX, Bleck CKE, Brinckmann J, Kozel BA. Copper-Binding Domain Variation in a Novel Murine Lysyl Oxidase Model Produces Structurally Inferior Aortic Elastic Fibers Whose Failure Is Modified by Age, Sex, and Blood Pressure. Int J Mol Sci. 2022; 23:6749. 10.3390/ijms23126749 35743192 PMC9223555

[34] Schräder CU, Heinz A, Majovsky P, Karaman Mayack B, Brinckmann J, Sippl W, Schmelzer CEH. Elastin is heterogeneously cross-linked. J Biol Chem. 2018; 293:15107–19. 10.1074/jbc.RA118.004322 30108173 PMC6166741

[35] Romier B, Ivaldi C, Sartelet H, Heinz A, Schmelzer CEH, Garnotel R, Guillot A, Jonquet J, Bertin E, Guéant JL, Alberto JM, Bronowicki JP, Amoyel J, et al. Production of Elastin-Derived Peptides Contributes to the Development of Nonalcoholic Steatohepatitis. Diabetes. 2018; 67:1604–15. 10.2337/db17-0490 29802129

[36] Bako G, Jacob MP, Fulop T Jr, Foris G, Leovey A, Robert L. Immunology of elastin: study of anti-elastin peptide antibodies by DOT immunobinding assay. Immunol Lett. 1987; 15:187–92. 10.1016/0165-2478(87)90023-x 3311975

[37] Frette C, Wei SM, Neukirch F, Sesboüé R, Martin JP, Jacob MP, Kauffmann F. Relation of serum elastin peptide concentration to age, FEV1, smoking habits, alcohol consumption, and protease inhibitor phenotype: an epidemiological study in working men. Thorax. 1992; 47:937–42. 10.1136/thx.47.11.937 1465752 PMC464101

[38] Paczek L, Michalska W, Bartlomiejczyk I. Trypsin, elastase, plasmin and MMP-9 activity in the serum during the human ageing process. Age Ageing. 2008; 37:318–23. 10.1093/ageing/afn039 18332058

[39] Paczek L, Michalska W, Bartlomiejczyk I. Proteolytic enzyme activity as a result of aging. Aging Clin Exp Res. 2009; 21:9–13. 10.1007/BF03324892 19225263

[40] Mancia G, Fagard R, Narkiewicz K, Redon J, Zanchetti A, Böhm M, Christiaens T, Cifkova R, De Backer G, Dominiczak A, Galderisi M, Grobbee DE, Jaarsma T, et al. 2013 ESH/ESC guidelines for the management of arterial hypertension: the Task Force for the Management of Arterial Hypertension of the European Society of Hypertension (ESH) and of the European Society of Cardiology (ESC). Eur Heart J. 2013; 34:2159–219. 10.1093/eurheartj/eht151 23771844

[41] Laurent S. Arterial stiffness: intermediate or surrogate endpoint for cardiovascular events? Eur Heart J. 2005; 26:1152–4. 10.1093/eurheartj/ehi280 15827059

[42] Node K, Kitakaze M, Yoshikawa H, Kosaka H, Hori M. Reduced plasma concentrations of nitrogen oxide in individuals with essential hypertension. Hypertension. 1997; 30:405–8. 10.1161/01.hyp.30.3.405 9314424

[43] Li P, Schmidt IM, Sabbisetti V, Tio MC, Opotowsky AR, Waikar SS. Plasma Endothelin-1 and Risk of Death and Hospitalization in Patients Undergoing Maintenance Hemodialysis. Clin J Am Soc Nephrol. 2020; 15:784–93. 10.2215/CJN.11130919 32381583 PMC7274287

[44] El-Eshmawy MM, El-Adawy EH, Mousa AA, Zeidan AE, El-Baiomy AA, Abdel-Samie ER, Saleh OM. Elevated serum neutrophil elastase is related to prehypertension and airflow limitation in obese women. BMC Womens Health. 2011; 11:1. 10.1186/1472-6874-11-1 21247478 PMC3031240

[45] Jing Y, Shi J, Lu B, Zhang W, Yang Y, Wen J, Hu R, Yang Z, Wang X. Association of Circulating Cathepsin S and Cardiovascular Disease Among Patients With Type 2 Diabetes: A Cross-Sectional Community-Based Study. Front Endocrinol (Lausanne). 2021; 12:615913. 10.3389/fendo.2021.615913 33746900 PMC7973458

[46] Skjøt-Arkil H, Clausen RE, Rasmussen LM, Wang W, Wang Y, Zheng Q, Mickley H, Saaby L, Diederichsen AC, Lambrechtsen J, Martinez FJ, Hogaboam CM, Han M, et al. Acute Myocardial Infarction and Pulmonary Diseases Result in Two Different Degradation Profiles of Elastin as Quantified by Two Novel ELISAs. PLoS One. 2013; 8:e60936. 10.1371/journal.pone.0060936 23805173 PMC3689773

[47] Ali K, Israr MZ, Ng LL, Mordi I, Lang CC, Kuzmanova E, Huang JT, Choy AM. Plasma desmosine for prediction of outcomes after acute myocardial infarction. Front Cardiovasc Med. 2022; 9:992388. 10.3389/fcvm.2022.992388 36479574 PMC9719937

[48] Mordi IR, Forsythe RO, Gellatly C, Iskandar Z, McBride OM, Saratzis A, Chalmers R, Chin C, Bown MJ, Newby DE, Lang CC, Huang JTJ, Choy AM. Plasma Desmosine and Abdominal Aortic Aneurysm Disease. J Am Heart Assoc. 2019; 8:e013743. 10.1161/JAHA.119.013743 31595818 PMC6818029

[49] Nilsson PM. Genetic and environmental determinants of early vascular ageing (EVA). Curr Vasc Pharmacol. 2012; 10:700–1. 10.2174/157016112803520981 23259558

[50] Li Y, Gray A, Xue L, Farb MG, Ayalon N, Andersson C, Ko D, Benjamin EJ, Levy D, Vasan RS, Larson MG, Rong J, Xanthakis V, et al. Metabolomic Profiles, Ideal Cardiovascular Health, and Risk of Heart Failure and Atrial Fibrillation: Insights From the Framingham Heart Study. J Am Heart Assoc. 2023; 12:e028022. 10.1161/JAHA.122.028022 37301766 PMC10356055

[51] Lloyd-Jones DM. Cardiovascular risk prediction: basic concepts, current status, and future directions. Circulation. 2010; 121:1768–77. 10.1161/CIRCULATIONAHA.109.849166 20404268

[52] Liew SM, Doust J, Glasziou P. Cardiovascular risk scores do not account for the effect of treatment: a review. Heart. 2011; 97:689–97. 10.1136/hrt.2010.220442 21474616 PMC3072200

[53] Bramwell JC, Hill AV. The velocity of the pulse wave in man. Proceedings of the royal society B. 1922; 93:298–306. 10.1098/rspb.1922.0022

[54] Brands PJ, Willigers JM, Ledoux LA, Reneman RS, Hoeks AP. A noninvasive method to estimate pulse wave velocity in arteries locally by means of ultrasound. Ultrasound Med Biol. 1998; 24:1325–35. 10.1016/s0301-5629(98)00126-4 10385955

[55] Maizel J, Six I, Slama M, Tribouilloy C, Sevestre H, Poirot S, Giummelly P, Atkinson J, Choukroun G, Andrejak M, Kamel S, Mazière JC, Massy ZA. Mechanisms of aortic and cardiac dysfunction in uremic mice with aortic calcification. Circulation. 2009; 119:306–13. 10.1161/CIRCULATIONAHA.108.797407 19118252

[56] Blaise S, Romier B, Kawecki C, Ghirardi M, Rabenoelina F, Baud S, Duca L, Maurice P, Heinz A, Schmelzer CE, Tarpin M, Martiny L, Garbar C, et al. Elastin-derived peptides are new regulators of insulin resistance development in mice. Diabetes. 2013; 62:3807–16. 10.2337/db13-0508 23919962 PMC3806616

[57] Nave AH, Mižíková I, Niess G, Steenbock H, Reichenberger F, Talavera ML, Veit F, Herold S, Mayer K, Vadász I, Weissmann N, Seeger W, Brinckmann J, Morty RE. Lysyl oxidases play a causal role in vascular remodeling in clinical and experimental pulmonary arterial hypertension. Arterioscler Thromb Vasc Biol. 2014; 34:1446–58. 10.1161/ATVBAHA.114.303534 24833797

[58] Doué M, Okwieka A, Berquand A, Gorisse L, Maurice P, Velard F, Terryn C, Molinari M, Duca L, Piétrement C, Gillery P, Jaisson S. Carbamylation of elastic fibers is a molecular substratum of aortic stiffness. Sci Rep. 2021; 11:17827. 10.1038/s41598-021-97293-5 34497312 PMC8426361

