# Supplementary material for: Exploring aortic stiffness in aging mice: a comprehensive methodological overview
Source: Aging (Albany NY). 2024 Dec 2;17(2):280–307. doi: 10.18632/aging.206168 (PMC11892926; doi:10.18632/aging.206168)
Supplement: Supplementary Materials [file aging-17-206168-s001.pdf]

SUPPLEMENTARY MATERIALS

SUPPLEMENTARY METHODS

Functional parameters

Measurement of blood pressure

Currently, multiple more or less invasive methods exist to study blood pressure in animals. In our case we have chosen the tail-cuff method, which is non-invasive and the closest to those measurements taken in humans. Other available methods require anesthesia and/or delicate surgery and are not suitable for the study of aortic remodeling. However, the tail-cuff method requires adaptation of the animals, handling, restraint and swelling of the sleeve of the Sphygmomanometer. Regularization is carried out every day, one week before the test. The final measurement is the average of five successive measurements as described in [1, 2]. Three parameters were analyzed, the pulse, the systolic (SBP) and diastolic (DBP) blood pressures. From these, the mean arterial pressure (MAP) and the pulsed pressure (PP) can be deduced from: PAM = 2/3 PAD + 1/3 PAS; PP = PAS – PAD. If the SBP and/or DBP of aged C57Bl/6 were statistically superior to the pressure of the young C57Bl/6 mice, then we considered the mice to be hypertensive.

Pulse wave velocity (PWV)

The velocity measurement is done in anesthetized animals (4% isoflurane, 10 min). The measurement can be made either from ultrasound probes (Indus Instruments, Webster, TX, USA), one placed at the level of the aortic arch and the other at the level of the abdominal thoracic aorta near the bifurcation of the iliac arteries. While the Doppler ultrasound system can measure a velocity limited to portions of arteries (carotids, aortic arch), this method considers the entire aorta and gives a more general idea of the rigidity of the vessel.

High-frequency ultrasound imaging

High resolution ultrasound imaging was performed with anesthetized animals (isoflurane 4%). The animals were depilated with hair removal cream and placed on a heated table (37°C). For ultrasound measurements, a Vevo3000 ultrasound imaging system (VisualSonics, Toronto, Canada) with a 30 MHz linear signal transducer was used for measurements of anatomical and functional parameters of left ventricle of heart needed to determine factors such as ejection fraction, cardiac output, fractional area change, fractional shortening, stroke volume, end-systolic volume (LVESV), and end-diastolic volume (LVEDV). At the level of the aortic arch, the diameter of the vessel was measured during the cardiac cycle (systole, Ds-diastole, Dd), as well as the pulse wave velocity (PWV) and the thickness of the tunica intima-media (h). Distensibility factor (DC) and Young's modulus (E) were derived by Bramwell and Hill [3] equation ($PWW = \sqrt{\frac{E \times h}{D \times \rho}}$) and Moens-Korteweg equation ($PWW = \sqrt{\frac{1}{\rho \times DC}}$), respectively. From the conclusions of Brands et al. [4], the local variation of the pressure during the cardiac cycle (DP) and the compliance (CC) can then be deduced as: $\Delta P = \frac{\frac{(As - Ad)}{Ad}}{DC}$ et $CC = \frac{(As - Ad)}{\Delta P}$. Additional information is available in the Supplementary Materials section.

Vascular reactivity

We investigated the effects of aging on *ex vivo* aortic reactivity. Wild-type mice were anesthetized by intraperitoneal injections of pentobarbital sodium (150 mg/kg). Once euthanasia was complete, their hearts were removed. Vascular reactivity studies were carried out, as described in [5]. A midline incision was made through the sternum to open up the thoracic cavity, and the descending thoracic aorta was carefully isolated. Each aorta was sectioned into 3.5 mm rings devoid of fat and connective tissue. The rings were placed in Kreb's-Henseleit (KH) solution under 5% CO_2 and 95% O_2 atmosphere at 37°C. The aorta rings were maintained under a 1.3 g tension (previously determined as the optimal point for their length-tension relationship) and allowed to equilibrate for 1 h. All rings were pre-constricted with potassium chloride (KCl). After rinsing, phenylephrine (between 10^{-9} and 3.10^{-5} mol/L) was added to the medium. The constriction was expressed as a percentage of the KCl response. The endothelium function was measured as the relaxation response to acetylcholine (between 10^{-9} and 3.10^{-5} mol/L). The degree of relaxation was calculated considering the maximal contraction obtained with phenylephrine.

Biochemical parameters

Cross-linking assay

Protein analysis was performed as described in [6]. Briefly, for collagen crosslink analysis, samples were reduced by sodium borohydride (Sigma, Germany; 25

mg $NaBH_4$/ml in 0.05 M NaH_2PO_4/0.15 M NaCl pH 7.4, 1 h on ice, 1.5 h at room temperature) and digested with high purity bacterial collagenase (C0773; Sigma, Germany; 50 U/ml, 37°C, 12 h). The soluble fractions containing collagen cross-links were hydrolyzed in 6 N HCl at 110°C for 24 h. The hydrolysates were precleared by solid phase extraction. Dried eluates were analyzed on an amino acid analyzer (Biochrom 30, Biochrom, Cambridge, UK). The nomenclature of the crosslinks used in the article refers to the reduced variants of crosslinks. The collagen content was analyzed in an aliquot of hydrolyzed samples of the collagenase soluble fraction prior to preclearance and calculated based on a content of 14 mg hydroxyproline in 100 mg collagen. For protein and elastin crosslinks analysis, samples were digested with bacterial collagenase [7]. The soluble fraction containing collagen was subjected to hydrolysis and amino acid analysis. The residual fraction was extracted by hot alkali (0.1 N NaOH, 95°C, 45 min). The supernatant containing non-collagenous/non-elastin proteins and the insoluble residue containing insoluble elastin were subjected to hydrolysis and amino acid analysis. The content of elastin crosslinks was analyzed in an aliquot of the NaOH-insoluble fraction containing elastin after CF-11 preclearance by amino acid analysis.

Gene expression

Was analyzed by qPCR, as previously described [20]. Total RNA was extracted using Trizol reagent (Eurobio Scientific, Les Ulis, France). The RNA concentration was measured using a NanoDrop system (Thermo Fisher Scientific, Illkirch, France). The 260/280 ratio was calculated, using NanoDrop software, to evaluate protein contamination. Complementary DNA (cDNA) was generated using a Verso cDNA kit (Thermo Fisher Scientific, Illkirch, France). Real-time PCR was performed using SYBR Green on a BioRad CFX96 Real-Time System (Bio-Rad, Hercules, CA, USA). In this study, 5 µl cDNA (1/10) and 0.7 µl of each forward and reverse primer (3 µM) were used for the qPCR test, with cycling conditions as follows: 95°C for 15 minutes, 40 cycles of 95°C for 10 seconds, and 60°C for 60 seconds. RNA expression was normalized to the housekeeping genes 36B4 and RPS26, and relative gene expression was calculated using the $2^{-\Delta\Delta CT}$ method. Supplementary Table 2 presents the forward and reverse sequences.

Imaging parameters

Raman spectroscopy/imaging

Raman measurements were performed with a Witec alpha 300R confocal Raman microscope (Witec GmbH, Ulm, Germany). Paraffin cross-sections (3 µm thickness) of aortas underwent deparaffinization by a consecutive row of xylene and ethanol steps and were rehydrated in PBS. Samples were kept hydrated during the entire measurement. For each sample, two images were acquired of an area of 80 × 90 µm, at a spatial resolution of 0.5 × 0.5 µm/pixel and an integration time of 0.05 s/spectrum. A green laser (532 nm) with an output power of 60 mW, a 600 g/mm grating and a 63× water dipping objective were selected for the measurements. Sections from 7 animals were measured for each group.

Data analysis

First, data were preprocessed by cosmic ray removal, baseline correction (shape algorithm), cropping to the wavenumber region between 300–3000 cm^{-1} and normalizing (area to 1 normalization). True component analysis (TCA, Witec Project 5.2 Software) was performed for image generation. Briefly, the TCA algorithm identifies most prevalent spectral signatures in the Raman maps, the corresponding pixel and thus allows to generate intensity distribution heatmaps for each component. For further in-depth analysis of molecular changes, single spectra were extracted from the preprocessed TCA images and analyzed by principal component analysis (PCA). PCA allows to decompose the spectral information to a defined number of vectors (principal components, PC), which elaborate spectral similarities and differences that can be explained by the corresponding loadings plot. PCA was performed for elastic fibers and the interfibrillar ECM. 400 spectra were extracted per animal and applied for PCA. Statistical analysis was performed by comparing the average score values of each animal (GraphPad Prism 9, unpaired t-test).

Atomic Force Microscopy (AFM)

Frozen 10 µm-thick aorta cross-sections were incubated in KH solution for equilibration at 37°C. The prepared samples were put onto the microscope stage and observed with bright field illumination to locate the spots of interest. Analysis was performed using AFM (Bioscope Catalyst, Bruker, Billerica, MA, USA, driven by the Nanoscope Analysis 1.8 software) coupled to a Nikon Eclipse Ti inverted microscope (Nikon, Tokyo, Japan). To obtain a representative set of values for each cross section, AFM analyses were performed at three different locations of the cross section, and each experiment was triplicated, leading to nine different areas analyzed per condition. Experiments were performed in the KH buffer using the Peak Force Quantitative NanoMechanical (PFQNM) mode with ScanAsyst-air probes (Bruker, Billerica, MA, USA) with a nominal spring constant of 0.4 N/m and a nominal resonant frequency of 70 kHz. For the

PFQNM calibration, the standard supplier protocol was applied to obtain quantitative measurements of the Young's modulus (YM). First, the deflection sensitivity was calibrated, before use in the buffer, by carrying out indentation ramps on a clean and hard sapphire surface. Then, the cantilever spring constant was calculated before and after each experiment, following the thermal tuning method. The last step was to calibrate the curvature radius of the tip using a standard titanium tip check sample. This curvature radius was confirmed by performing a test measurement of the YM of a calibrated known sample. A PeakForce frequency of 0.25 kHz was used to maximize the contact time between the tip and the sample, and the PeakForce amplitude was set to 2 µm. The distance synchronization parameter was manually and constantly adjusted over time so that the turnaway point of each force curve was exactly at the (x, y) maximum position. Images were captured with a resolution of 256 pixels per line. Once the different AFM images were acquired, the force curves were extracted from chosen areas in the PFQNM images for the YM calculation, and the conventional Derjaguin–Muller–Toporov (DMT) model was used to fit the linear part of the extension curve, as it was identified as the best suited model according to the tip geometry and the properties of the samples. The YM at each point of the elastic fibers or of the inter-fiber spaces was calculated using a value of the Poisson ratio of 0.5 for our samples considered incompressible. For each condition, at least 5000 force curves were treated to obtain the mean values of the YM for the elastic fibers and the inter-fiber spaces. The analyses were performed at three different locations in each cross-section, for a total of nine cross-sections obtained from three different mice.

High-resolution X-ray microscopy

Sample preparation

Aorta specimens from 6-month-old mice (n = 4) and 20-months-old mice (n = 4) were received embedded in paraffin. The paraffin was removed by immersion in xylene for 30 min, followed by staining with a 0.5% I_2 in ethanol solution for 30 min. After immersion in xylene once again for 30 min, the sample was embedded in paraffin and, using a heated blade, it was manually cut into sections of ca 500 µm thickness orthogonal to the aorta longest axis. Afterwards, the sample was glued onto the tip of a metallic pin with the aid of a stereo microscope.

X-ray imaging

A Carl Zeiss Xradia 810 Ultra X-ray microscope equipped with a chromium source (5.4 keV) was used in the imaging experiments. The sample located onto the tip of a metallic pin was inserted in the sample holder of the device and the experiments were performed using Zernike phase-contrast. Samples were scanned using a field-of-view of 64 μm^2. A total of 901 projection images, with an exposure time of 20 s each, were acquired by rotating the sample over 180°. Each sample was imaged for two or more times, and the reconstructed volumetric images were stitched after reconstruction. Image reconstruction was performed by a filtered back-projection algorithm using the XMReconstructor software integrated into the Xradia 810 Ultra and the final images have isotropic voxel size of 128 nm. The tomograms obtained were exported as a stack of 16-bit TIFF images for stitching and visualization in Thermo Fischer Avizo software (version 3D 2021.1).

Scanning electron microscopy

Defrosted samples were deposited onto a SEM stub and treated with NanoSuit® Aqueous Solution (Electron Microscopy Sciences) according to the manufacturer instructions. Samples were imaged in a Scanning Electron Microscope FEI Quanta 3D FEG Dual-Beam working at an acceleration voltage of 5 kV.

SUPPLEMENTARY REFERENCES

1. Romier B, Dray C, Vanalderwiert L, Wahart A, Hocine T, Dortignac A, Garbar C, Garbar C, Boulagnon C, Bouland N, Maurice P, Bennasroune A, Sartelet H, et al. Apelin expression deficiency in mice contributes to vascular stiffening by extracellular matrix remodeling of the aortic wall. Sci Rep. 2021; 11:22278. https://doi.org/10.1038/s41598-021-01735-z PMID:34782679

2. Berquand A, Wahart A, Henry A, Gorisse L, Maurice P, Blaise S, Romier-Crouzet B, Pietrement C, Bennasroune A, Sartelet H, Jaisson S, Gillery P, Martiny L, et al. Revealing the elasticity of an individual aortic fiber during ageing at nanoscale by in situ atomic force microscopy. Nanoscale. 2021; 13:1124–33. https://doi.org/10.1039/d0nr06753a PMID:33399602

3. Bramwell JC. The velocity of the pulse wave in man. Proceedings of the royal society B. 1922; 93: 298–306.

4. Brands PJ, Willigers JM, Ledoux LA, Reneman RS, Hoeks AP. A noninvasive method to estimate pulse wave velocity in arteries locally by means of ultrasound. Ultrasound Med Biol. 1998; 24:1325–35. https://doi.org/10.1016/s0301-5629(98)00126-4 PMID:10385955

5. Maizel J, Six I, Slama M, Tribouilloy C, Sevestre H, Poirot S, Giummelly P, Atkinson J, Choukroun G,

Andrejak M, Kamel S, Mazière JC, Massy ZA. Mechanisms of aortic and cardiac dysfunction in uremic mice with aortic calcification. Circulation. 2009; 119:306–13.
https://doi.org/10.1161/CIRCULATIONAHA.108.797407
PMID:19118252

6. Nave AH, Mižíková I, Niess G, Steenbock H, Reichenberger F, Talavera ML, Veit F, Herold S, Mayer K, Vadász I, Weissmann N, Seeger W, Brinckmann J, Morty RE. Lysyl oxidases play a causal role in vascular remodeling in clinical and experimental pulmonary arterial hypertension. Arterioscler Thromb Vasc Biol. 2014; 34:1446–58.
https://doi.org/10.1161/ATVBAHA.114.303534
PMID:24833797

7. Leray C, Pelletier X, Hemmendinger S, Cazenave JP. Thin-layer chromatography of human platelet phospholipids with fatty acid analysis. J Chromatogr. 1987; 420:411–6.
https://doi.org/10.1016/0378-4347(87)80198-6
PMID:3693512

8. Henry JP, Meehan JP, Stephens P, Santisteban GA. Arterial pressure in cba mice as related to age. J Gerontol. 1965; 20:239–43.
https://doi.org/10.1093/geronj/20.2.239
PMID:14284803

9. Pezet M, Jacob MP, Escoubet B, Gheduzzi D, Tillet E, Perret P, Huber P, Quaglino D, Vranckx R, Li DY, Starcher B, Boyle WA, Mecham RP, Faury G. Elastin haploinsufficiency induces alternative aging processes in the aorta. Rejuvenation Res. 2008; 11:97–112.
https://doi.org/10.1089/rej.2007.0587
PMID:18173368

10. Mariko B, Pezet M, Escoubet B, Bouillot S, Andrieu JP, Starcher B, Quaglino D, Jacob MP, Huber P, Ramirez F, Faury G. Fibrillin-1 genetic deficiency leads to pathological ageing of arteries in mice. J Pathol. 2011; 224:33–44.
https://doi.org/10.1002/path.2840
PMID:21432852

11. Elias MF, Pentz CA 3rd. Blood pressure extremes and activity in aging mice. Physiol Behav. 1977; 19:811–3.
https://doi.org/10.1016/0031-9384(77)90320-1
PMID:609621

12. Sonesson B, Hansen F, Stale H, Länne T. Compliance and diameter in the human abdominal aorta--the influence of age and sex. Eur J Vasc Surg. 1993; 7:690–7.
https://doi.org/10.1016/s0950-821x(05)80718-2
PMID:8270073

13. Lakatta EG, Levy D. Arterial and cardiac aging: major shareholders in cardiovascular disease enterprises: Part II: the aging heart in health: links to heart disease. Circulation. 2003; 107:346–54.
https://doi.org/10.1161/01.cir.0000048893.62841.f7
PMID:12538439

14. Lakatta EG, Levy D. Arterial and cardiac aging: major shareholders in cardiovascular disease enterprises: Part I: aging arteries: a "set up" for vascular disease. Circulation. 2003; 107:139–46.
https://doi.org/10.1161/01.cir.0000048892.83521.58
PMID:12515756

15. Lakatta EG. Arterial and cardiac aging: major shareholders in cardiovascular disease enterprises: Part III: cellular and molecular clues to heart and arterial aging. Circulation. 2003; 107:490–7.
https://doi.org/10.1161/01.cir.0000048894.99865.02
PMID:12551876

16. Greenwald SE. Ageing of the conduit arteries. J Pathol. 2007; 211:157–72.
https://doi.org/10.1002/path.2101
PMID:17200940

17. Jadidi M, Habibnezhad M, Anttila E, Maleckis K, Desyatova A, MacTaggart J, Kamenskiy A. Mechanical and structural changes in human thoracic aortas with age. Acta Biomater. 2020; 103:172–88.
https://doi.org/10.1016/j.actbio.2019.12.024
PMID:31877371

18. Kawel-Boehm N, Hetzel SJ, Ambale-Venkatesh B, Captur G, Francois CJ, Jerosch-Herold M, Salerno M, Teague SD, Valsangiacomo-Buechel E, van der Geest RJ, Bluemke DA. Reference ranges ("normal values") for cardiovascular magnetic resonance (CMR) in adults and children: 2020 update. J Cardiovasc Magn Reson. 2020; 22:87.
https://doi.org/10.1186/s12968-020-00683-3
PMID:33308262

19. Gharraee N, Sun Y, Swisher JA, Lessner SM. Age and sex dependency of thoracic aortopathy in a mouse model of Marfan syndrome. Am J Physiol Heart Circ Physiol. 2022; 322:H44–56.
https://doi.org/10.1152/ajpheart.00255.2021
PMID:34714692

20. Wheeler JB, Mukherjee R, Stroud RE, Jones JA, Ikonomidis JS. Relation of murine thoracic aortic structural and cellular changes with aging to passive and active mechanical properties. J Am Heart Assoc. 2015; 4:e001744.
https://doi.org/10.1161/JAHA.114.001744
PMID:25716945

21. Hemmeryckx B, Hoylaerts MF, Deloose E, Van Hove CE, Fransen P, Bult H, Lijnen HR. Age-associated pro-inflammatory adaptations of the mouse thoracic aorta. Thromb Haemost. 2013; 110:785–94.

https://doi.org/10.1160/TH13-01-0022 PMID:23925372

22. Cavinato C, Murtada SI, Rojas A, Humphrey JD. Evolving structure-function relations during aortic maturation and aging revealed by multiphoton microscopy. Mech Ageing Dev. 2021; 196:111471. https://doi.org/10.1016/j.mad.2021.111471 PMID:33741396

23. Sokolis DP. Time-course of axial residual strain remodeling and layer-specific thickening during aging along the human aorta. J Biomech. 2020; 112:110065. https://doi.org/10.1016/j.jbiomech.2020.110065 PMID:33035841

24. Kelleher CM, McLean SE, Mecham RP. Vascular extracellular matrix and aortic development. Curr Top Dev Biol. 2004; 62:153–88. https://doi.org/10.1016/S0070-2153(04)62006-0 PMID:15522742

25. Fritze O, Romero B, Schleicher M, Jacob MP, Oh DY, Starcher B, Schenke-Layland K, Bujan J, Stock UA. Age-related changes in the elastic tissue of the human aorta. J Vasc Res. 2012; 49:77–86. https://doi.org/10.1159/000331278 PMID:22105095

26. Liu SL, Bae YH, Yu C, Monslow J, Hawthorne EA, Castagnino P, Branchetti E, Ferrari G, Damrauer SM, Puré E, Assoian RK. Matrix metalloproteinase-12 is an essential mediator of acute and chronic arterial stiffening. Sci Rep. 2015; 5:17189. https://doi.org/10.1038/srep17189 PMID:26608672

27. Hornebeck W, Adnet JJ, Robert L. Age dependent variation of elastin and elastase in aorta and human breast cancers. Exp Gerontol. 1978; 13:293–8. https://doi.org/10.1016/0531-5565(78)90037-2 PMID:738376

28. McNulty M, Spiers P, McGovern E, Feely J. Aging is associated with increased matrix metalloproteinase-2 activity in the human aorta. Am J Hypertens. 2005; 18:504–9. https://doi.org/10.1016/j.amjhyper.2004.11.011 PMID:15831360

29. Leloup AJ, Van Hove CE, Heykers A, Schrijvers DM, De Meyer GR, Fransen P. Elastic and Muscular Arteries Differ in Structure, Basal NO Production and Voltage-Gated Ca(2+)-Channels. Front Physiol. 2015; 6:375. https://doi.org/10.3389/fphys.2015.00375 PMID:26696904

30. Ng HH, Jelinic M, Parry LJ, Leo CH. Increased superoxide production and altered nitric oxide-mediated relaxation in the aorta of young but not old male relaxin-deficient mice. Am J Physiol Heart Circ Physiol. 2015; 309:H285–96. https://doi.org/10.1152/ajpheart.00786.2014 PMID:25957220

31. Al-Shaer MH, Choueiri NE, Correia ML, Sinkey CA, Barenz TA, Haynes WG. Effects of aging and atherosclerosis on endothelial and vascular smooth muscle function in humans. Int J Cardiol. 2006; 109:201–6. https://doi.org/10.1016/j.ijcard.2005.06.002 PMID:16054252

32. Andrawis N, Jones DS, Abernethy DR. Aging is associated with endothelial dysfunction in the human forearm vasculature. J Am Geriatr Soc. 2000; 48:193–8. PMID:10682949

33. Blackwell KA, Sorenson JP, Richardson DM, Smith LA, Suda O, Nath K, Katusic ZS. Mechanisms of aging-induced impairment of endothelium-dependent relaxation: role of tetrahydrobiopterin. Am J Physiol Heart Circ Physiol. 2004; 287:H2448–53. https://doi.org/10.1152/ajpheart.00248.2004 PMID:15319209

34. Novella S, Dantas AP, Segarra G, Vidal-Gómez X, Mompeón A, Garabito M, Hermenegildo C, Medina P. Aging-related endothelial dysfunction in the aorta from female senescence-accelerated mice is associated with decreased nitric oxide synthase expression. Exp Gerontol. 2013; 48:1329–37. https://doi.org/10.1016/j.exger.2013.08.003 PMID:23948180

35. Donato AJ, Magerko KA, Lawson BR, Durrant JR, Lesniewski LA, Seals DR. SIRT-1 and vascular endothelial dysfunction with ageing in mice and humans. J Physiol. 2011; 589:4545–54. https://doi.org/10.1113/jphysiol.2011.211219 PMID:21746786

36. Gerhard M, Roddy MA, Creager SJ, Creager MA. Aging progressively impairs endothelium-dependent vasodilation in forearm resistance vessels of humans. Hypertension. 1996; 27:849–53. https://doi.org/10.1161/01.hyp.27.4.849 PMID:8613259

37. Taddei S, Virdis A, Mattei P, Ghiadoni L, Fasolo CB, Sudano I, Salvetti A. Hypertension causes premature aging of endothelial function in humans. Hypertension. 1997; 29:736–43. https://doi.org/10.1161/01.hyp.29.3.736 PMID:9052889

38. Huynh J, Nishimura N, Rana K, Peloquin JM, Califano JP, Montague CR, King MR, Schaffer CB, Reinhart-King CA. Age-related intimal stiffening enhances endothelial permeability and leukocyte

transmigration. Sci Transl Med. 2011; 3:112ra122. https://doi.org/10.1126/scitranslmed.3002761 PMID:22158860

39. Lee K, Saidel GM, Penn MS. Permeability change of arterial endothelium is an age-dependent function of lesion size in apolipoprotein E-null mice. Am J Physiol Heart Circ Physiol. 2008; 295:H2273–9. https://doi.org/10.1152/ajpheart.00242.2008 PMID:18835923

40. Carlson KH, Bourne WM, McLaren JW, Brubaker RF. Variations in human corneal endothelial cell morphology and permeability to fluorescein with age. Exp Eye Res. 1988; 47:27–41. https://doi.org/10.1016/0014-4835(88)90021-8 PMID:3409985

41. Krettek A, Sukhova GK, Libby P. Elastogenesis in human arterial disease: a role for macrophages in disordered elastin synthesis. Arterioscler Thromb Vasc Biol. 2003; 23:582–7. https://doi.org/10.1161/01.ATV.0000064372.78561.A5 PMID:12615674

42. Docherty JR, O'Malley K. Ageing and alpha-adrenoceptors. Clin Sci (Lond). 1985; 68:133s–36. https://doi.org/10.1042/cs068s133 PMID:2857609

43. Elliott HL, Sumner DJ, McLean K, Reid JL. Effect of age on the responsiveness of vascular alpha-adrenoceptors in man. J Cardiovasc Pharmacol. 1982; 4:388–92. https://doi.org/10.1097/00005344-198205000-00008 PMID:6177934

44. Fenton M, Huang HL, Hong Y, Hawe E, Kurz DJ, Erusalimsky JD. Early atherogenesis in senescence-accelerated mice. Exp Gerontol. 2004; 39:115–22. https://doi.org/10.1016/j.exger.2003.10.004 PMID:14724071

45. Li Y, Gilbert TR, Matsumoto AH, Shi W. Effect of aging on fatty streak formation in a diet-induced mouse model of atherosclerosis. J Vasc Res. 2008; 45:205–10. https://doi.org/10.1159/000112133 PMID:18063868

46. Simo OK, Berrougui H, Fulop T, Khalil A. The Susceptibility to Diet-Induced Atherosclerosis Is Exacerbated with Aging in C57B1/6 Mice. Biomedicines. 2021; 9:487. https://doi.org/10.3390/biomedicines9050487 PMID:33946646

47. Tyrrell DJ, Blin MG, Song J, Wood SC, Zhang M, Beard DA, Goldstein DR. Age-Associated Mitochondrial Dysfunction Accelerates Atherogenesis. Circ Res. 2020; 126:298–314. https://doi.org/10.1161/CIRCRESAHA.119.315644 PMID:31818196

48. Yagi K, Komura S, Sasaguri Y, Yoshino K, Ohishi N. Atherogenic change in the thoracic aorta of the senescence-accelerated mouse. Atherosclerosis. 1995; 118:233–6. https://doi.org/10.1016/0021-9150(95)05609-2 PMID:8770317

49. Fernández-Friera L, Peñalvo JL, Fernández-Ortiz A, Ibañez B, López-Melgar B, Laclaustra M, Oliva B, Mocoroa A, Mendiguren J, Martínez de Vega V, García L, Molina J, Sánchez-González J, et al. Prevalence, Vascular Distribution, and Multiterritorial Extent of Subclinical Atherosclerosis in a Middle-Aged Cohort: The PESA (Progression of Early Subclinical Atherosclerosis) Study. Circulation. 2015; 131:2104–13. https://doi.org/10.1161/CIRCULATIONAHA.114.014310 PMID:25882487

50. Paydary K, Revheim ME, Emamzadehfard S, Gholami S, Pourhassan S, Werner TJ, Høilund-Carlsen PF, Alavi A. Quantitative thoracic aorta calcification assessment by ^{18}F-NaF PET/CT and its correlation with atherosclerotic cardiovascular disorders and increasing age. Eur Radiol. 2021; 31:785–94. https://doi.org/10.1007/s00330-020-07133-9 PMID:32870396
