# Supplementary material for: Exploring aortic stiffness in aging mice: a comprehensive methodological overview
Source: Aging (Albany NY). 2024 Dec 2;17(2):280–307. doi: 10.18632/aging.206168 (PMC11892926; doi:10.18632/aging.206168)
Supplement: Supplementary Figures [file aging-17-206168-s002.pdf]

SUPPLEMENTARY FIGURES

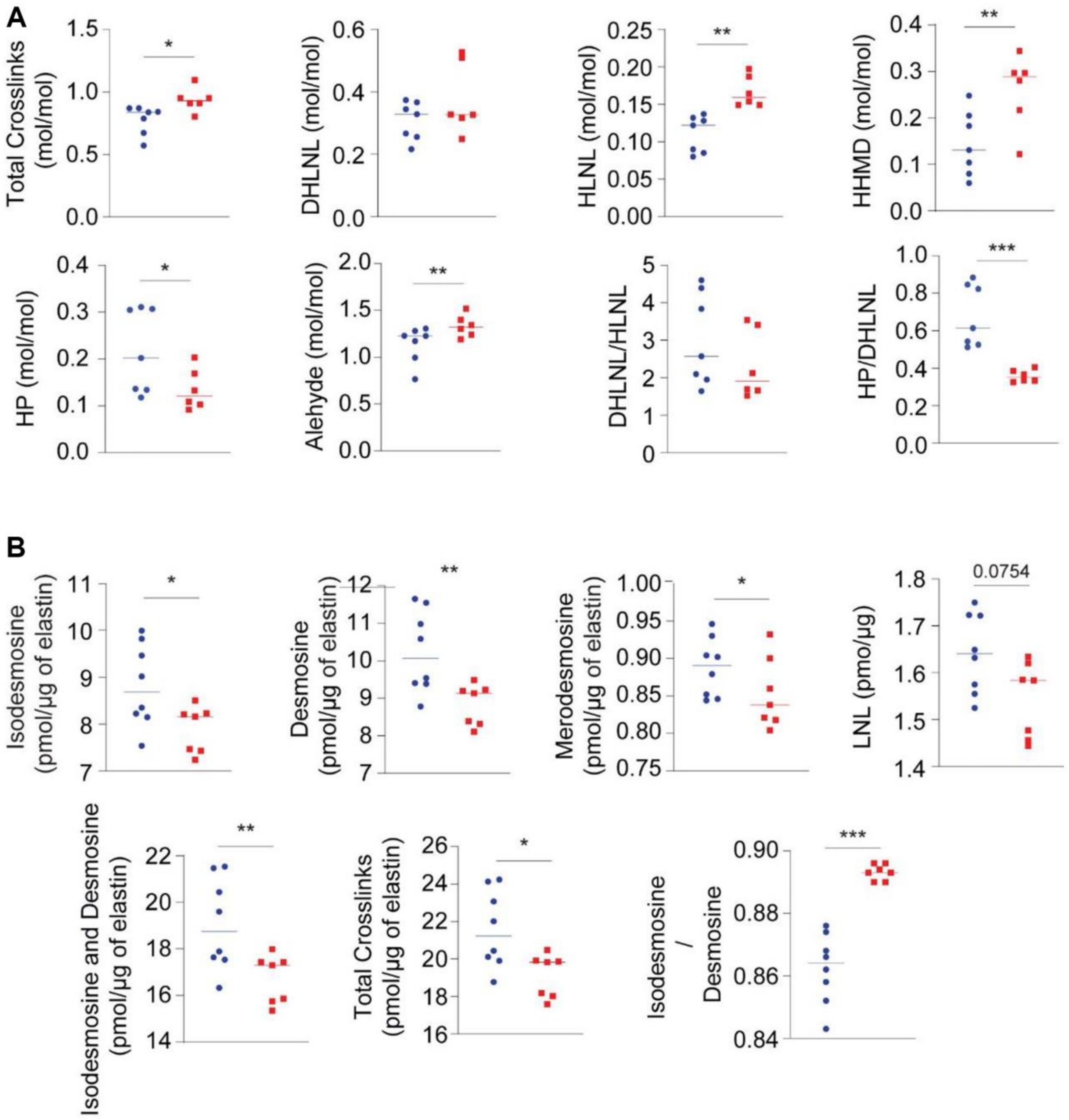

Supplementary Figure 1. Evaluation of crosslinks in the abdominal aorta of young (blue) or old (red) mice. (**A**) Collagen cross-links. (**B**) Elastin cross-links. Statistical test: Mann-Whitney. Mean +/− SEM. Significant differences ($^{*}p$ < 0.05, $^{**}p$ < 0.001, $^{***}p$ < 0.0001, Mann-Whitney).

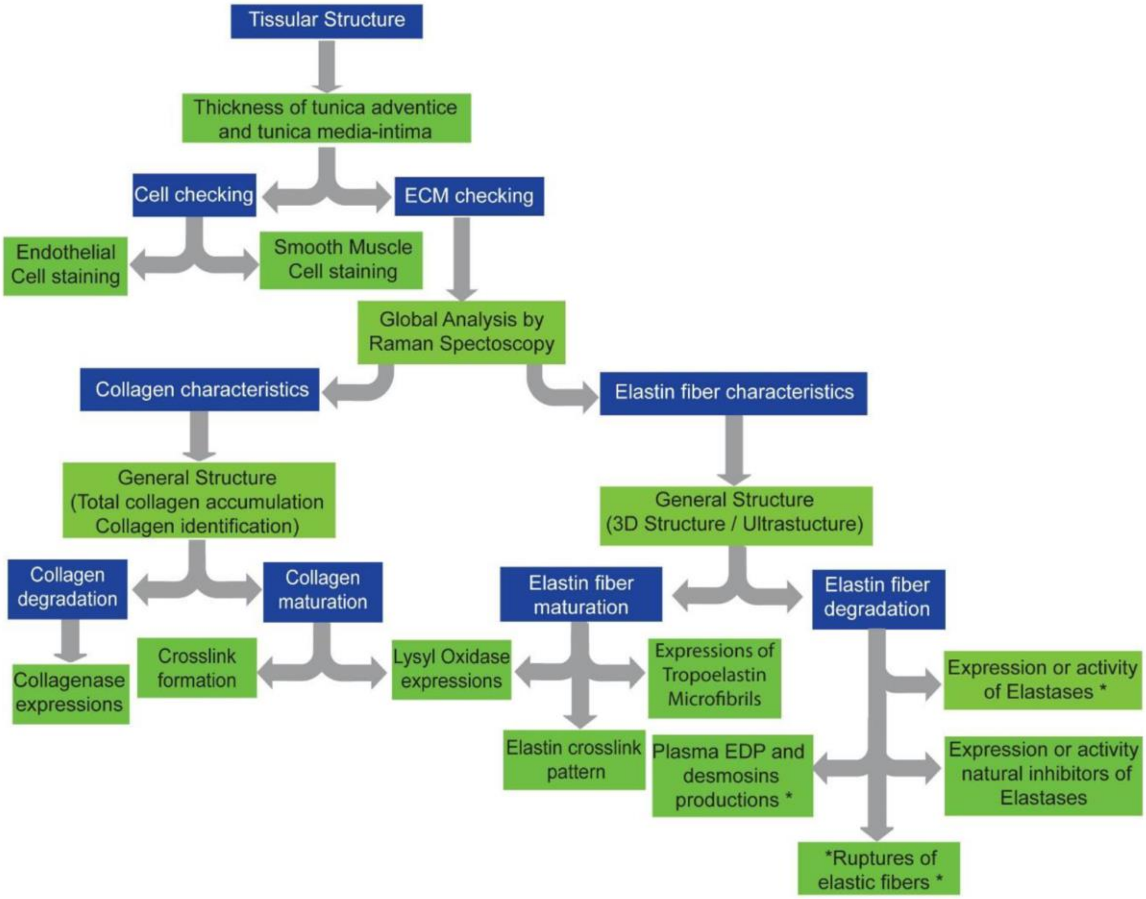

Supplementary Figure 2. Summary of the decision tree allowing the study of vascular stiffness by morphological approaches. "*" identifies parameters that are methodologically accessible for clinical studies.
