# Supplementary material for: Exploring aortic stiffness in aging mice: a comprehensive methodological overview
Source: Aging (Albany NY). 2024 Dec 2;17(2):280–307. doi: 10.18632/aging.206168 (PMC11892926; doi:10.18632/aging.206168)
Supplement: Supplementary Tables [file aging-17-206168-s003.pdf]

SUPPLEMENTARY TABLES

Supplementary Table 1. Variations of aortic parameters from young to aged in mice and humans.

	Mouse	Human
**Systolic blood pressure (mmHg)**	= or ↑ [8–11]	↑ [12, 13]
**Aortic stiffness**	↑ [9, 10, 14]	↑ [12, 13, 15–18]
**Aortic structure**		
Lumen diameter	↑ [9, 10, 19, 21]	↑ [12, 15, 16, 18]
Wall thickness	↑ [9, 10, 20, 21]	↑ [14–17]
Number of elastic lamellae	= [9, 10]	= [17]
Adventitia thickness	= [22]	= [23]
**Elastic fiber-related processes in the aortic wall**		
Elastogenesis	↓ [24]	↓ [25]
Elastolysis	↑ [26]	= or ↑ [25, 27, 28]
**Endothelial cell function**		
Basal NO production	↓ [21, 29, 30]	↓ (Dilation of forearm arteries) [31, 32]
Acetylcholine-triggered NO production	↓ [21, 30, 33, 34]	↓ (Dilation of forearm arteries) [15, 35–37]
Permeability	↑ [38, 39]	↑ (Corneal endothelial cells) [40]
**Vascular smooth muscle cell function**		
Elastin production	↓ [24]	↓ [25, 41]
Contractility	↓, = or ↑ [9, 10, 20, 21, 34]	↓, = or ↑ [42, 43]
**Susceptibility to atherogenesis**	↑ or = [44–48]	↑ [13, 49, 50]
**Left ventricular hypertrophy (LVH)**	↑ [9]	↑ [13]

The complete list of references (here in brackets) can be found in the supplementary section.

Supplementary Table 2. Sequences used for qPCR.

Gene name	Forward sequence (5′ → 3′)	Reverse sequence (5′ → 3′)
RPS26	TAGAAGCCGCTGCTGTCAGG	GGCACAGCTCACGCAATAATG
36B4	AAAGCCTGGAAGAAGGAGGTC	AGATTCGGGATATGCTGTTGG
MMP-1	CACTCCCTTGGGCTCACTCA	GTTGCACCTGTTGGCTGGAT
MMP-2	ATCGAGACCATGCGGAAGC	GCCCGAGCAAAAGCATCAT
MMP-8	AACGGGAAGACATAC	GGGTCCATGGATCTT
MMP-9	CACGGAGACGGGTATCCCTT	GGGCACCATTTGAGTTTCCAT
MMP13	CAGTCTCCGAGGAGAAACTATGAT	GGACTTTGTCAAAAAGAGCTCAG
Myosin light-chain kinase (*MLCK*)	TGGGGGACGTGAAACTGTTTG	GGGGCAGAATGAAAGCTGG
Neutrophil elastase (NE)	TGGAGGTCATTTCTGTGGTG	CTGCACTGACCGGAAATTTAG
Cathepsin-S	GCGTCACTGAGGTGAAATACC	CCCCCACAGCACTGAAAG
Cystatin C	ATGACCAGCCCCATCTGA	CCAGGGCACGCTGTAGAT
Tissue inhibitors of metalloproteinase (Timp1)	TCCCCAGAAATCAACGAGACC	GTACCGGATATCTGCGGCATT
Serine peptidase inhibitor (SERPIN)	TAGGGAGCAAGGGTGACACTC	ACTGTCTGGTCTGTTGAGGGT
Elastin (ELN)	GCTGCTGCTAAGGCTGCTAA	AGCACCTGGGAGCCTAACTC
Fibrillin 1 (FBN1)	GGACGGAAAGAACTGTGAAGAT	ACACATTCCGTTTAGGCACA
Fibulin 5 (FBLN5)	ATCTGCTGATTGGTGAAAACC	ATGGTGAATGGCTGGTCTCT
Lysyl oxidase like 1 (LOXL1)	GAATACTGAGCCAGACTGGC	GGGTCTCATTGAAATTAGTATCC
Lysyl oxidase like 2 (LOXL2)	ATGACCTGCTGAGCCTCAAC	CAGTGTCCTCCAGGCAGAAG
Lysyl oxidase like 3 (LOXL3)	ATGGGTGCCATCCACTTGAG	TGTTCTTGGACGGGCATCTC
Lysyl oxidase like 4 (LOXL4)	GGTTGTGAACCCCACAAACG	CTGCATTGGCTCGGTAGGAA

Lysyl oxidase (LOX)	CTATTCGATCCCACGCTGCT	CCTCACAATGGGGATGTGCT
Latent TGF beta binding protein (Ltbp-4)	CGTCAACGAGTGTGATGAGG	GAGCAAATCCTGGACGACAG
Smooth muscle actin (α—SMA)	ACTGGTATTGTGCTGGACTCTG	TAGTCACGAAGGAATAGCCACG
Smooth muscle (SM22α)	CCCAGACACCGAAGCTACTC	GACTGCACTTCTCGGCTCAT
h-caldesmon	TACACCAATGCAATCGAGGGAA	TACATCTCCTGGCCTCAAGTCA
